# Target Site Recognition by a Diversity-Generating Retroelement

**DOI:** 10.1371/journal.pgen.1002414

**Published:** 2011-12-15

**Authors:** Huatao Guo, Longping V. Tse, Angela W. Nieh, Elizabeth Czornyj, Steven Williams, Sabrina Oukil, Vincent B. Liu, Jeff F. Miller

**Affiliations:** 1 Department of Microbiology, Immunology, and Molecular Genetics, David Geffen School of Medicine, University of California Los Angeles, Los Angeles, California, United States of America; 2 AvidBiotics Corporation, South San Francisco, California, United States of America; 3 The Molecular Biology Institute, University of California Los Angeles, Los Angeles, California, United States of America; Agency for Science, Technology, and Research, Singapore

## Abstract

Diversity-generating retroelements (DGRs) are *in vivo* sequence diversification machines that are widely distributed in bacterial, phage, and plasmid genomes. They function to introduce vast amounts of targeted diversity into protein-encoding DNA sequences via mutagenic homing. Adenine residues are converted to random nucleotides in a retrotransposition process from a donor template repeat (TR) to a recipient variable repeat (VR). Using the *Bordetella* bacteriophage BPP-1 element as a prototype, we have characterized requirements for DGR target site function. Although sequences upstream of VR are dispensable, a 24 bp sequence immediately downstream of VR, which contains short inverted repeats, is required for efficient retrohoming. The inverted repeats form a hairpin or cruciform structure and mutational analysis demonstrated that, while the structure of the stem is important, its sequence can vary. In contrast, the loop has a sequence-dependent function. Structure-specific nuclease digestion confirmed the existence of a DNA hairpin/cruciform, and marker coconversion assays demonstrated that it influences the efficiency, but not the site of cDNA integration. Comparisons with other phage DGRs suggested that similar structures are a conserved feature of target sequences. Using a kanamycin resistance determinant as a reporter, we found that transplantation of the IMH and hairpin/cruciform-forming region was sufficient to target the DGR diversification machinery to a heterologous gene. In addition to furthering our understanding of DGR retrohoming, our results suggest that DGRs may provide unique tools for directed protein evolution via *in vivo* DNA diversification.

## Author Summary

Diversity-generating retroelements function through a unique, reverse transcriptase–mediated “copy and replace” mechanism that enables repeated rounds of protein diversification, selection, and optimization. The ability of DGRs to introduce targeted diversity into protein-coding DNA sequences has the potential to dramatically accelerate the evolution of adaptive traits. The utility of these elements in nature is underscored by their widespread distribution throughout the bacterial domain. Here we define DNA sequences and structures that are necessary and sufficient to direct the diversification machinery to specified target sequences. In addition to providing mechanistic insights into conserved features of DGR activity, our results provide a blueprint for the use of DGRs for a broad range of protein engineering applications.

## Introduction

Diversity-generating retroelements (DGRs) have been identified in numerous bacterial phyla [1], [2]. Although most DGRs are bacterial chromosomal elements, they are prevalent in phage and plasmid genomes as well. The prototype DGR was identified in a temperate bacteriophage, BPP-1, on the basis of its ability to switch tropism for different receptor molecules on host *Bordetella* species [3]. Tropism switching is mediated by a phage-encoded DGR which introduces nucleotide substitutions in a gene that specifies a host cell-binding protein, Mtd (major tropism determinant), positioned at the distal tips of phage tail fibers. This allows phage adaptation to the dynamic changes in cell surface molecules that occur during the infectious cycle of its bacterial host [3]. Comparative bioinformatics predicts that all DGRs function by a fundamentally similar mechanism using conserved components ([1]; Gingery et al., unpublished data). These include unique reverse transcriptase (RT) genes (*brt* for BPP-1), accessory loci (*avd* or *HRDC*), short DNA repeats, and target genes that are specifically diversified [1]–[4].

**Figure 1:**
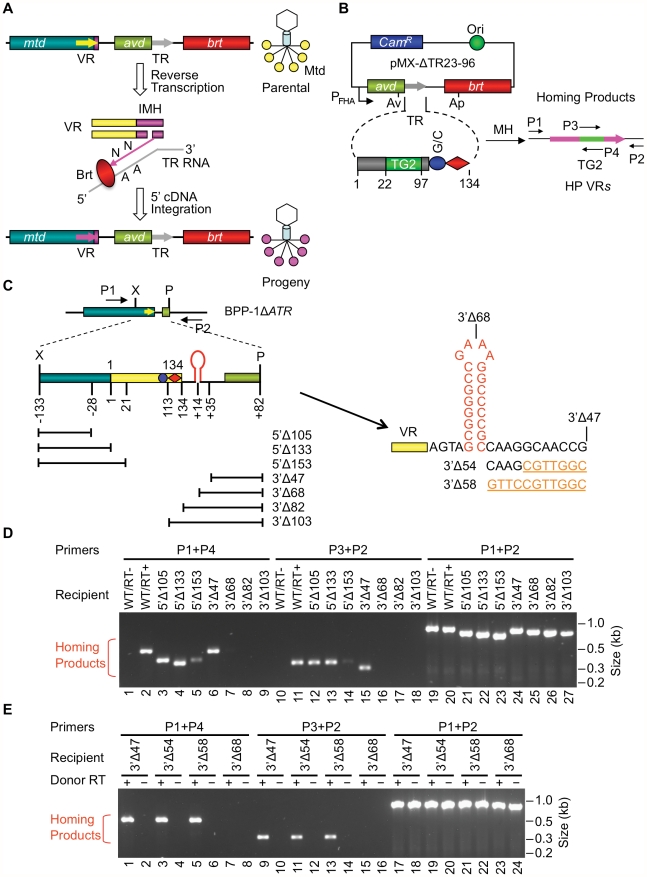
Boundaries of the BPP-1 DGR target sequence. (A) Tropism switching by *Bordetella* phage BPP-1 is mediated by its DGR through mutagenic homing, which is proposed to occur through a target DNA-primed reverse transcription (TPRT) mechanism [4]. *mtd*, *avd*, *brt*, the variable and template repeats (VR and TR) are indicated. VR diversification leads to altered Mtd trimers at the distal tail fibers of progeny phages. (B) PCR-based DGR homing assay. Plasmid pMX-ΔTR23–96 carries the BPP-1 *avd*-TR-*brt* region placed downstream of the BvgAS-regulated P_fha_ promoter and contains a 30 bp insert (TG2) between TR positions 22 and 97 [4]. Grey and pink arrows represent TR and VR, respectively. Small horizontal arrows indicate primers used for homing assays: P1 and P2 are sense- and antisense-strand primers annealing upstream and downstream of VR, respectively; P3 and P4, sense- and antisense-strand primers, respectively, that anneal to TG2. *Cam^R^*, chloramphenicol resistance gene. (C) BPP-1 DGR target region and deletion constructs. 5′ or 3′ deletions start from position −133 upstream of VR or position +82 downstream of VR, respectively. Lines below the target represent regions deleted. The region downstream of VR contains two 8 bp inverted repeats, which can potentially form a hairpin/cruciform structure. Constructs 3′Δ54 and 3′Δ58 were derived from 3′Δ47 by changing 7 and 11 residues downstream of the hairpin/cruciform structure to their complementary residues, respectively, and were assayed in E. (D) PCR-based DGR homing assays showed that BPP-1 target recognition does not require any sequence upstream of VR, but does require up to 35 bp downstream of VR, which includes the potential hairpin/cruciform-forming region. Donor RT+indicates pMX-ΔTR23–96, - indicates pMX-ΔTR23–96 with the SMAA mutation. (E) Fine mapping of the 3′ boundary of the BPP-1 DGR target with additional deletion constructs 3′Δ54 and 3′Δ58 showed that no sequences downstream of position +24 are required for target recognition.

As illustrated by the BPP-1 DGR shown in Figure 1A, diversity results from the introduction of nucleotide substitutions in a variable repeat (VR) located at the 3′ end of the *mtd* gene [1]–[4]. Variable sites in VR correspond to adenine residues in a homologous template repeat (TR), which remains unchanged throughout the process [1]–[4]. Transcription of TR provides an essential RNA intermediate that is reverse transcribed by Brt, creating a cDNA product which ultimately replaces the parental VR [4]. During this unidirectional retrotransposition process of mutagenic homing, TR adenines are converted to random nucleotides which subsequently appear at corresponding positions in VR [1]–[4]. Adenine mutagenesis appears to occur during cDNA synthesis and is likely to be an intrinsic property of the DGR-encoded RT [4].

Located at the 3′ end of VR is the IMH (initiation of mutagenic homing) region, which consists of at least two functional elements: a 14 bp GC-only sequence [(GC)_14_] which is identical to the corresponding segment of TR, and a 21 bp sequence containing 5 mismatches with TR that determines the directionality of information transfer [1]. Using a saturating co-conversion assay, we have precisely mapped a marker transition boundary that appears to represent the point at which 3′ cDNA integration occurs and information transfer begins [4]. This maps within the (GC)_14_ element and we previously postulated that it represents the site of a nick or double-strand break in the target DNA [4]. If true, the resulting 3′ hydroxyl could serve to prime reverse transcription of the TR-derived RNA intermediate in a target DNA-primed reverse transcription (TPRT) mechanism [4]–[7]. cDNA integration at the 5′ end of VR requires TR/VR homology and may occur via template switching during cDNA synthesis [4].

There are 23 adenines upstream of the (GC)_14_ element in the BPP-1 TR, each of which is capable of variation [3]. The theoretical maximum DNA sequence diversity is ∼10^14^, which translates to a maximum protein diversity of nearly 10 trillion distinct polypeptides at the C-terminus of Mtd. For Mtd and other DGR-diversified proteins, co-evolution has resulted in the precise positioning of TR adenines to correspond to solvent exposed residues in the ligand binding pockets of variable proteins [8], [9]. As implicated in Figure 1A, mutagenic homing occurs through a “copy and replace” mechanism that precisely regenerates all *cis*-acting components required for further rounds of diversification [4]. This allows the system to operate over and over again to optimize ligand-receptor interactions.

The goal of this study was to characterize requirements for target site recognition by the BPP-1 DGR. Along with insights into the mechanism of mutagenic homing, our results reveal engineering principles that allow DGRs to be exploited to diversify heterologous genes through a process that is entirely contained within bacterial cells.

## Results

### Boundaries of the BPP-1 DGR target sequence

5′ and 3′ boundaries of the BPP-1 DGR target sequence were delineated using a PCR-based assay that specifically detects VR sequences that have been modified by DGR-mediated retrohoming [4]. The system consists of a donor plasmid (pMX-ΔTR23-96, Figure 1B) carrying *avd*, a modified TR containing a 30 bp tag (TG2), and *brt* co-expressed from a BvgAS-regulated promoter [4], and a recipient prophage genome deleted for *avd*, TR, and *brt* (BPP-1Δ*ATR*, Figure 1C). TR retrotransposition from the donor plasmid to the recipient prophage VR creates a “tagged” VR that can be detected using primer pairs specific for the tag and VR-flanking sequences (P1/P4 and P2/P3 in Figure 1B; Table 1). Controls include the demonstration that homing products are Brt-dependent and contain mutagenized adenines. An advantage of this assay is that it does not require infectious phage particle formation and consequently allows manipulation of sequences that are required for Mtd function.

**Table 1:** List of oligonucleotides used in this study.

Name	Sequence
P1	5′ TTCGGTACCTGCTAGGCGTCAACCACCTG
P2	5′ AGCAAGCTTGTCCTGTTTGCGCGTGATGCT
P3	5′ AAATCTAGATCTGTCTGCGTTTGTGTT
P4	5′ AGCAAGCTTAGCACAGGAACACAAACG
P5	5′ GGTCACCATGAGCATTTGGTCGTAGCA
P6	5′ GTACAGCGGGCCGTCGTTCTCGTTCGCGTT
P7	5′ CCCTCTAGAGCTCCGGTTGCTTGTGGACG
P8	5′ AGCAAGCTTCCTCGATGGGTTCCAT
P9	5′ ATATCTAGACGTTTTCTTGGGTCTACCGTTTAATGTCG
P10	5′ ATAAAGCTTCGACATTAAACGGTAGACCCAAGAAAA

Deletions were introduced into VR and adjacent sequences in BPP-1Δ*ATR* lysogens (Figure 1C and Figure S1) and the abilities of mutated prophages to serve as recipients in retrohoming assays were measured (see Materials and Methods). As shown in Figure 1D, sequences upstream of VR were dispensable for DGR homing (lanes 4&13). A deletion mutation that truncates the first 20 bp of VR still supported homing, although at a decreased level (lanes 5&14). Sequence analysis of homing products for this mutant suggested that 5′ cDNA integration occurred at cryptic sites within the truncated VR, although 3′ cDNA integration occurred in a normal manner (Figure 1D, lanes 5&14; Figure S2). At the 3′ end, homing was highly dependent on a 35 bp region located downstream of VR (lanes 6&15 vs. lanes 7&16). This implicated sequences with 8 bp inverted repeats that could potentially form a hairpin structure in ssDNA or a cruciform structure in dsDNA as a possible determinant of DGR target function (Figure 1C). Additional analysis showed that deletion of sequences immediately downstream of the stem was well tolerated (3′Δ58, Figure 1C and 1E), while further deletions at the 3′ end (3′Δ68) reduced target function to essentially non-detectable levels in homing assays.

In the experiments in Figure 1, homing products were not detected using a donor plasmid expressing enzymatically inactive Brt (BrtSMAA, in which the active site motif YADD is replaced by SMAA; [1], [3], [4]), and sequence analysis of products generated with primer sets P1/P4 and P2/P3 demonstrated transfer of the TG2 tag from TR to VR. Adenine mutagenesis was observed in ∼53% of clones containing P1/P4 products and ∼32% of clones containing P2/P3 products (data not shown), which had 3 and 2 TR adenine residues available for mutagenesis, respectively. These observations indicated that true DGR homing products were being detected. Equivalent amounts of template phage DNA, as measured by quantitative PCR, were included in each experiment (lanes 19–27, Figure 1D; lanes 17–24, Figure 1E).

### Stem structure, but not sequence, is critical for DGR homing and phage tropism switching

**Figure 2:**
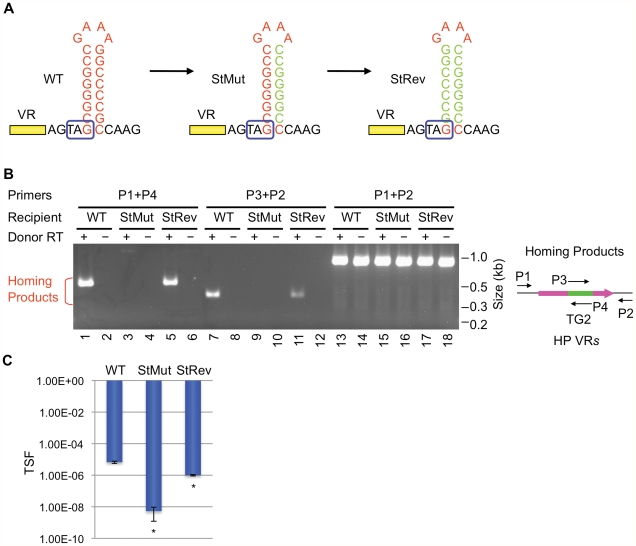
The hairpin/cruciform structure downstream of VR is required for target recognition. (A) WT and mutant hairpin/cruciform structures. Boxed TAG, *mtd* stop codon. (B) The hairpin/cruciform structure downstream of VR, as opposed to the primary sequence of the stem, is required for DGR mutagenic homing. PCR-based DGR homing assays are shown on the left, with primers used to detect homing products shown on the right. Sequence analysis of homing products demonstrated TG2 transfer from TR to VR with WT (data not shown) and StRev recipients (Figure S3). Adenine mutagenesis was observed in 7/12 of WT and 8/15 of StRev homing products amplified with primers P1 and P4, and 6/10 of WT and 5/20 of StRev homing products amplified with P2 and P3 (data not shown and Figure S3). (C) The hairpin/cruciform structure downstream of VR is required for phage tropism switching. The graph shows phage tropism switching frequencies (TSF) for BPP-1Δ*ATR* with WT or mutant hairpin/cruciform structures. The bars represent mean TSF ± standard deviations (s.d.). P values comparing mutants to WT in Student's t tests are indicated with asterisks. *P<0.02.

We next determined whether the primary sequence or the secondary structure of the putative hairpin/cruciform located downstream of VR is important for function. To disrupt the structure, 7 consecutive residues proximal to the loop on the 3′ half of the stem were changed to their complementary residues (StMut, Figure 2A). The resulting mutant was essentially unable to support DGR homing at a level that could be detected in PCR-based assays (lanes 3&9, Figure 2B). Complementary substitutions were subsequently introduced to the 5′ half of the stem to generate StRev (Figure 2A). If the primary sequence is important, the StRev recipient should remain non-functional. Alternatively, if the structure of the stem is the critical element, restoring base pairing interactions might restore DGR target function. As shown in Figure 2B (lanes 5&11), this appears to be the case, as the StRev mutant regained DGR homing activity. Homing products were verified by sequencing and adenine mutagenesis was observed (Figure S3).

Phage tropism switching assays provide a quantitative measure of DGR function [1], [3], [4]. Although the evolution of new ligand specificities is an inherently stochastic process, the frequency at which it occurs reflects the combined efficiencies of retrohoming and adenine mutagenesis. In Figure 2C, tropism switching was measured using BPP-1Δ*ATR* or mutant derivatives complemented with plasmid pMX1, which provides *avd*, TR and *brt* in *trans* (see Materials and Methods). The StMut mutation resulted in over a 1000-fold decrease in tropism switching, which was restored to near WT levels by the StRev allele. Sequence analysis of VR regions in phages with switched tropisms (5 random clones each) confirmed adenine mutagenesis in every case (Figures S4, S5, S6).

Taken together, these data argue that the ability to form a hairpin or cruciform structure, as opposed to the primary sequence of the inverted repeats, is a critical determinant of target site recognition. The residual tropism switching activity of StMut phage suggests that hairpin/cruciform-independent pathways may exist, although they operate at a much lower efficiency.

### Physical evidence for hairpin/cruciform formation in negatively supercoiled DNA

**Figure 3:**
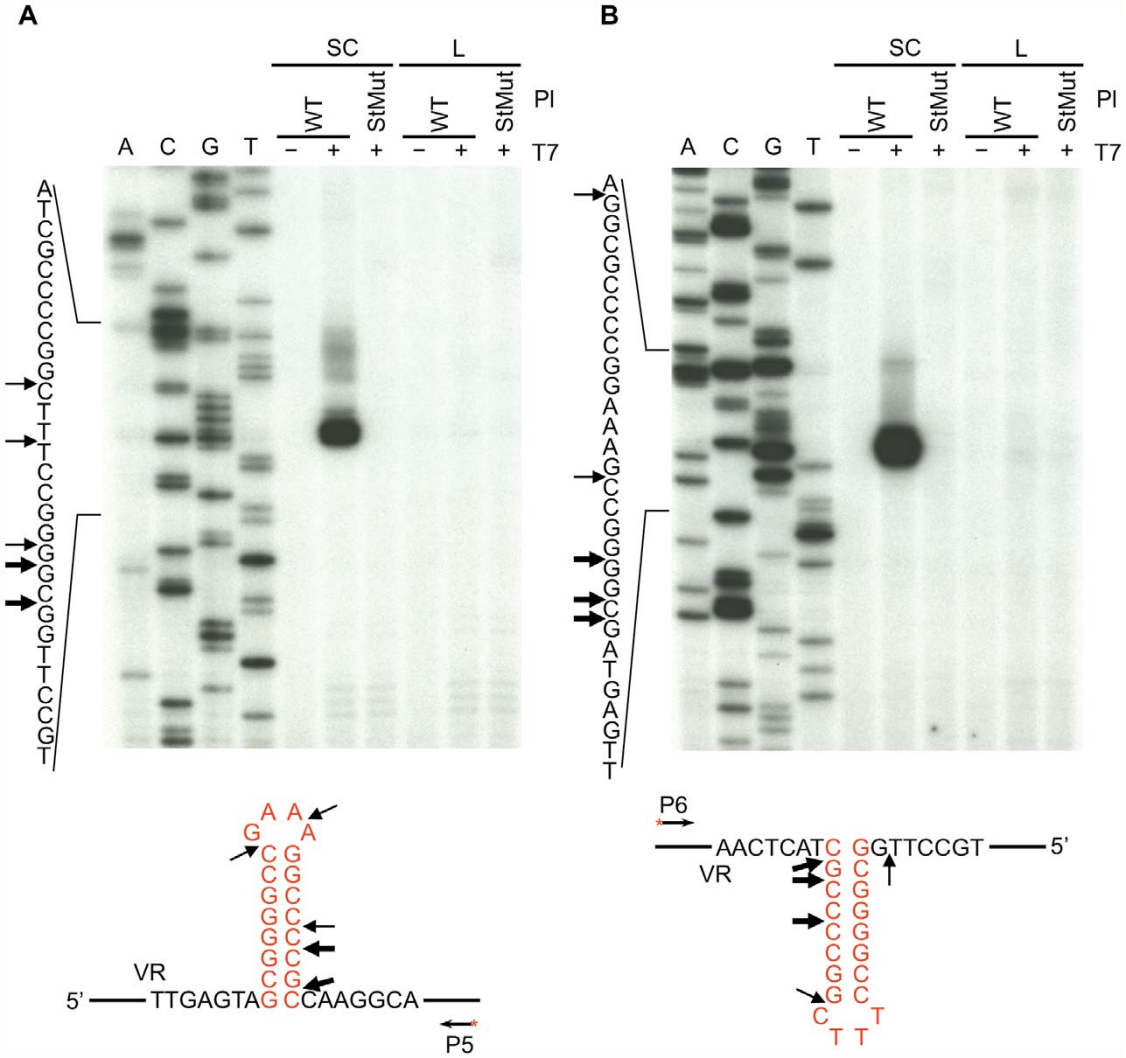
Hairpin/cruciform structure formation in negatively supercoiled DNA. Primer extension assays were used to identify T7 endonuclease I cleavage sites in plasmid DNA containing either the WT or StMut target sequences. (A) Top strand cleavage, (B) bottom strand cleavage. Supercoiled or linearized plasmid DNA was either left untreated (−) or digested with T7 endonuclease I (+) followed by primer extension. Sequence ladders are shown to the left and primer extension termination sites in supercoiled WT plasmid DNA are shown underneath the gels. Thick and thin arrows designate major and minor cleavage sites, respectively. The exact positions of cleavage sites are +/−2–3 nt due to uncertainty resulting from compression of the sequence ladder in the hairpin/cruciform region caused by DNA secondary structure. The heavily labeled major products on both strands represent multiple adjacent cleavage sites which were resolved at lower exposures. The figure is representive of results obtained from multiple independent experiments. P5 and P6 (Table 1) are the 5′ end-labeled primers used for primer extensions in A and B, respectively. SC, supercoiled plasmids; L, linearized plasmids; Pl, plasmid; T7, T7 endonuclease I.

To determine if the hairpin/cruciform structure can form *in vitro*, supercoiled plasmids carrying WT or mutant BPP-1 DGR target sequences were isolated and treated with phage T7 DNA endonuclease I, followed by primer extension with 5′ end-labeled primers to identify specific cleavage sites [10], [11]. T7 DNA endonuclease I is a structure-specific enzyme that resolves DNA four-way (Holiday) junctions and has previously been used to identify DNA hairpin or cruciform formation [10], [11]. As shown in Figure 3, cleavage sites were detected on both DNA strands in the hairpin/cruciform structure, with major cleavage sites at or near the four-way junction. Minor cleavage sites were also detected at or near the loop, as T7 DNA endonuclease I also has some activity on single-stranded DNA [12]. T7 endonuclease I cleavage at the hairpin/cruciform region requires structure formation, as plasmids containing a disrupted stem (StMut) were not cleaved in the corresponding region. Linearization of plasmids containing the WT sequence eliminated cleavage, suggesting that negative supercoiling is required for hairpin/cruciform formation [13], [14]. These results demonstrate that hairpins can form on either strand of the target DNA. Although it is likely that they form simultaneously on both strands to create cruciforms, this is not directly addressed by enzyme cleavage assays, hence the hairpin/cruciform designation.

### The BPP-1 DGR target sequence functions in an orientation-independent manner

**Figure 4:**
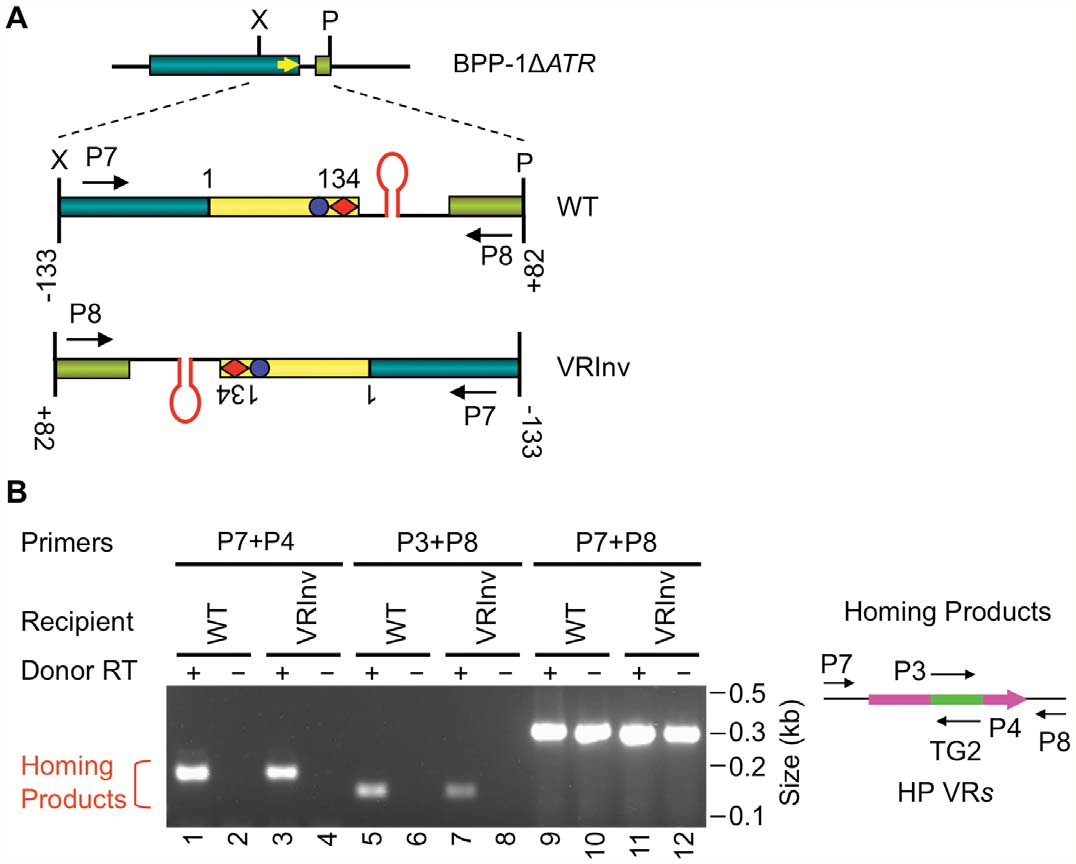
Target orientation in the BPP-1 phage genome is not critical for recognition. (A) Schematic of the BPP-1Δ*ATR* phage genome with WT or inverted (VRInv) target sequences. A segment from position −133 upstream of VR to position +82 downstream of VR was inverted. VR-flanking primers used in the DGR homing assay are also indicated. (B) Target inversion had no significant effect on DGR homing activity. pMX-ΔTR23–96 or its RT-deficient derivative were used as donors for the indicated recipients and products from PCR-homing assays are shown. The diagram to the right shows primers used for the assay. Sequence analysis of VRInv homing products showed TG2 transfer from TR to VR, as well as adenine mutagenesis in 5/10 homing products amplified with primers P7 (Table 1) and P4, and 2/9 products amplified with P8 (Table 1) and P3 (Figure S7).

We next determined whether the orientation of the target sequence relative to the phage genome is important for DGR retrohoming. In the experiment in Figure 4A, a segment of the BPP-1Δ*ATR* prophage that includes VR and its flanking sequences was inverted, and PCR-based DGR homing assays were performed with donor plasmid pMX-ΔTR23-96. DGR homing into the inverted target occurred at a level comparable to that of the WT control (Figure 4B), and sequence analysis indicated that normal homing products were produced (Figure S7). These results show that the polarity of phage replication is not important for DGR homing, and that the hairpin/cruciform structure functions in a manner that is independent of its orientation relative to the leading or lagging strands formed during DNA replication.

### Conservation and functional characteristics of DGR hairpin/cruciform structures

**Figure 5:**
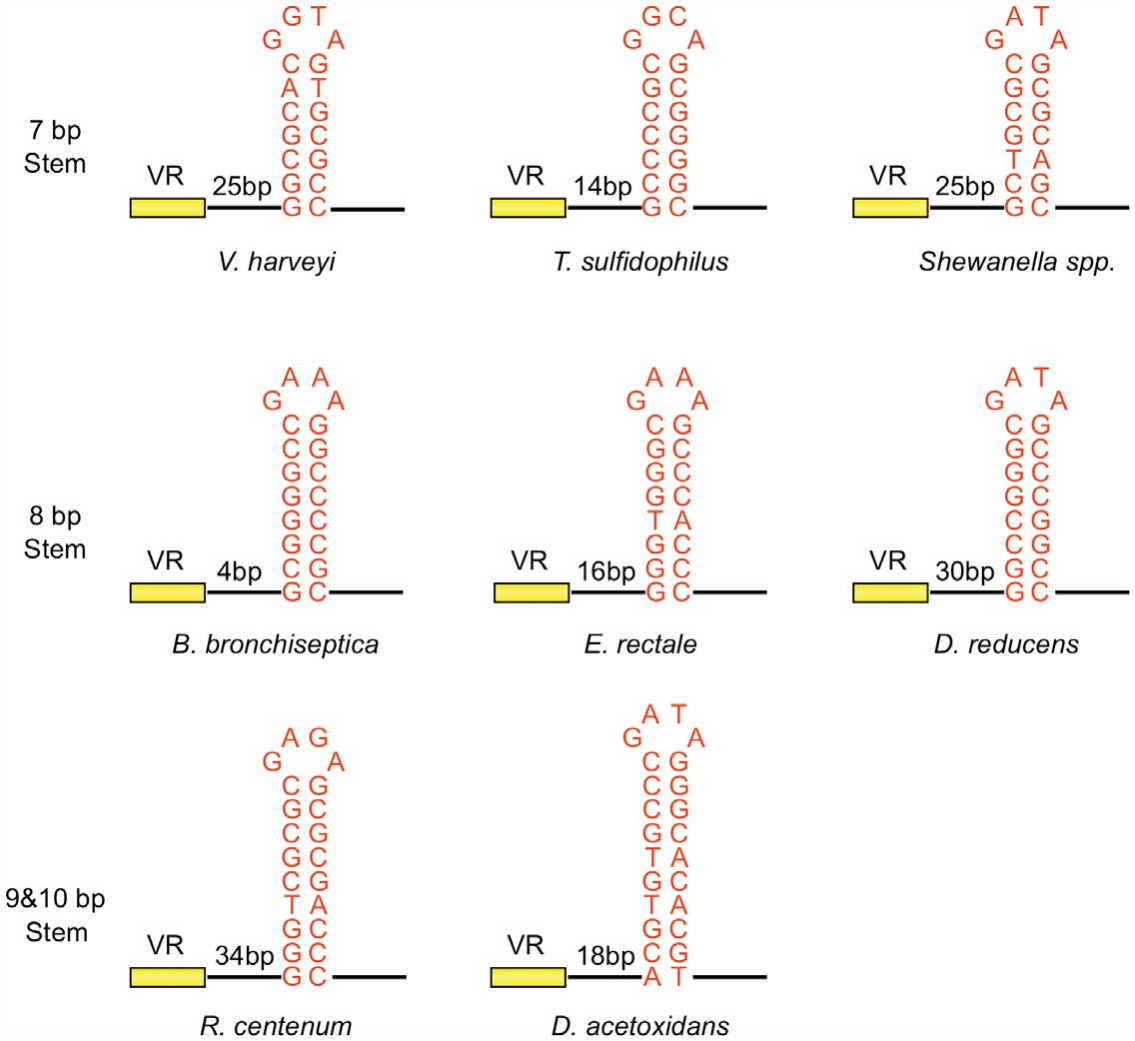
Phage-related DGRs contain potential hairpin/cruciform structures with conserved features. DGRs associated with phages or phage-related sequences in different bacterial genomes were identified as described in Doulatov *et al.* [1]. Short inverted repeats which could potentially form hairpin/cruciform structures were found downstream of VRs as shown. In each case, GC-rich stems are 7–10 bp in length with 4 nt loops composed of the conserved sequence [5′GRNA; R = A or G, N = any nucleotide]. Relative distances between the hairpin/cruciform structures and their corresponding VRs range from 4 to 34 bp. *V. harveyi*, *Vibrio harveyi* phage VHML; *T. sulfidophilus*, *Thioalkalivibrio sulfidophilus* HL-EbGr7; *Shewanella sp.*, *Shewanella sp.* W3-18-1; *B. bronchiseptica*, *Bordetella bronchiseptica* phage BPP-1; *E. rectale*, *Eubacterium rectale* DSM 17629; *D. reducens*, *Desulfotomaculum reducens* MI-1; *R. centenum*, *Rhodospirillum centenum* SW; *D. acetoxidans*, *Desulfotomaculum acetoxidans* DSM 771.

Inverted repeats are nearly always found downstream of VR sequences in target genes [Gingery et al., unpublished data], as illustrated by the phage DGR sequences shown in Figure 5. These elements display a striking pattern of similarity, suggesting they have conserved and important functions. In each case, hairpin/cruciform structures with 7–10 bp GC-rich stems and 4 nt loops can potentially be formed. Although stems are always GC-rich, their sequences differ, while loops are more conserved with the consensus sequence (5′GRNA3′, with R = A or G, N = any nucleotide) in the sense strand. The exact distance between the hairpin/cruciform structures and the 3′ ends of their respective VRs appears to be quite flexible. We took advantage of the BPP-1 DGR system to test the relevance of these patterns of conservation, with the goal of generating a more comprehensive understanding of parameters important for target site recognition.

**Figure 6:**
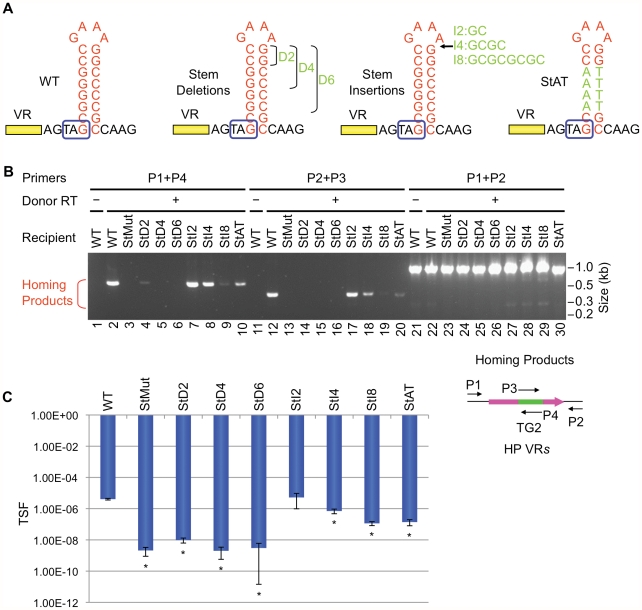
The stem length and sequence are important but can tolerate minor modifications. (A) Modifications in the stem of the hairpin/cruciform structure are shown. StD2, StD4 and StD6 are deletion mutants; StI2, StI4 and StI8 are insertion mutants. StAT has four GC base pairs in the middle of the stem changed to AT base pairs. Boxed TAG, *mtd* stop codon. (B) The stem is important for BPP-1 DGR mutagenic homing but can tolerate minor changes. PCR-homing assays are shown along with primers used. (C) Effects of stem modifications on phage tropism switching frequencies. The scale bars represent mean TSF ± s.d. P values comparing mutants to WT in Student's t tests are indicated with asterisks. *P<0.02.

**Figure 7:**
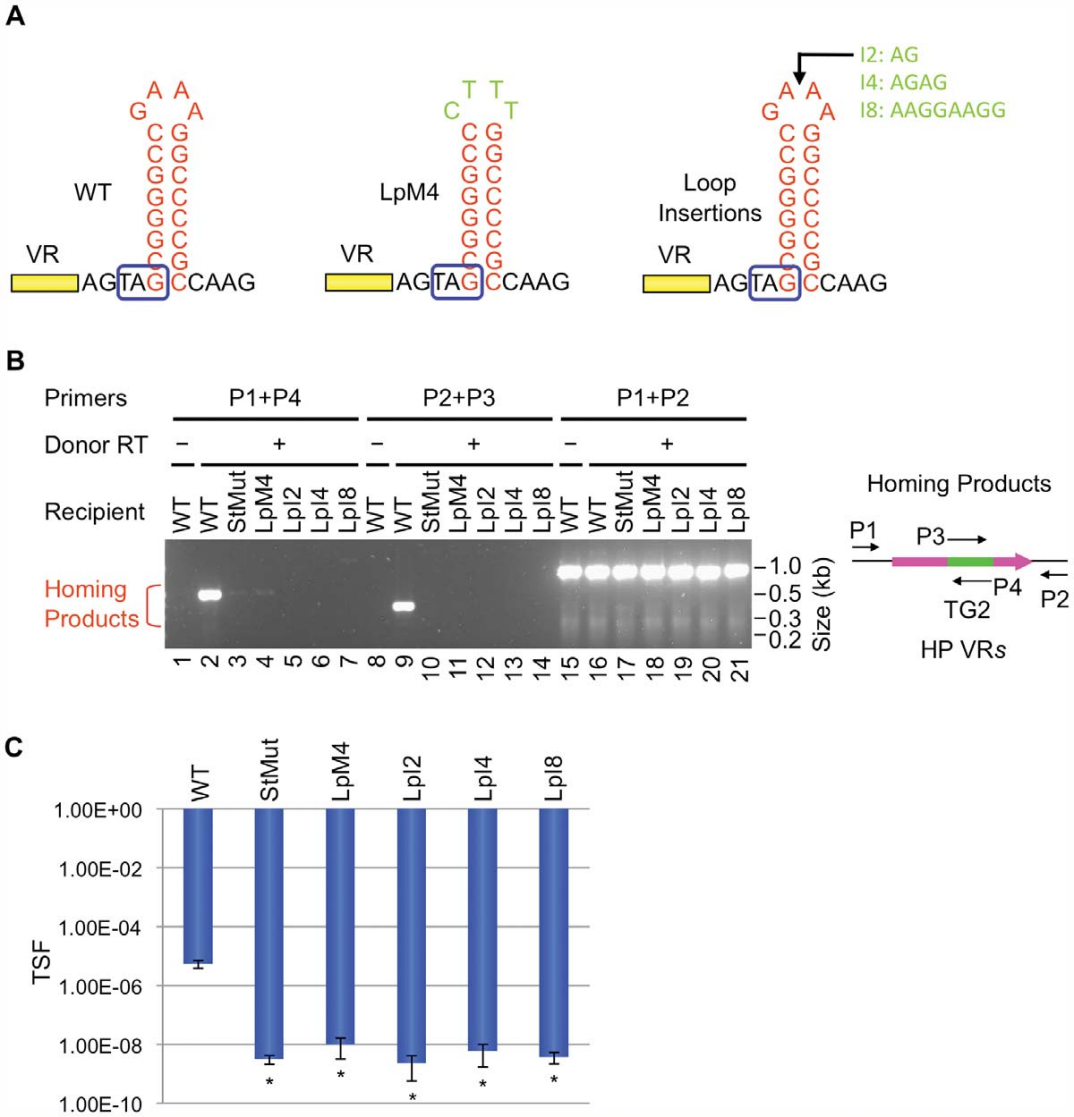
The loop of the hairpin/cruciform structure is critical for DGR function. (A) Substitution mutations or insertions in loop sequences are shown. Boxed TAG, *mtd* stop codon. (B) Both the loop sequence and size are highly critical for DGR mutagenic homing. PCR-homing assays are shown along with primers used. (C) Effects of loop modifications on phage tropism switching frequencies. The bars represent mean TSF ± s.d. P values comparing mutants to WT in Student's t tests are indicated with asterisks. *P<0.05.

We first studied requirements for stem length and sequence and found that although minor changes are tolerated, the WT configuration appears to be optimized for BPP-1 DGR function. Of the stem length variants in Figure 6A, extensions are better tolerated than deletions. Removal of 2, 4 or 6 bp proximal to the loop results in markedly decreased activity in both PCR-based homing (Figure 6B) and phage tropism switching assays (Figure 6C), to levels similar to those observed with the StMut allele in which the stem is completely abolished (Figure 2A). Insertion of 2 bp next to the loop had little effect on activity, while longer insertions gradually decreased target site function. Keeping the length of the stem constant, a sequence change in the middle of the stem that converts 4 GC base pairs to AT base pairs (StAT, Figure 6A) greatly reduced, but did not eliminate function. We next tested the effects of altering the sequence and size of the loop using the mutant constructs shown in Figure 7A. Substituting CTTT for the consensus loop sequence GAAA, or increasing the size of the loop by as little as 2 nt, decreased activity in PCR-homing (Figure 7B) and tropism switching assays (Figure 7C) to near background levels. Based on these experiments, it appears that an 8–10 bp GC-rich stem is optimal for BPP-1 DGR homing, and that both the size and sequence of the 4 bp loop are critical for function. Our results correlate with the patterns of conservation shown in Figure 5.

### Shifting the position of the hairpin/cruciform element alters the efficiency of target site recognition but not the site of 3′ cDNA integration

**Figure 8:**
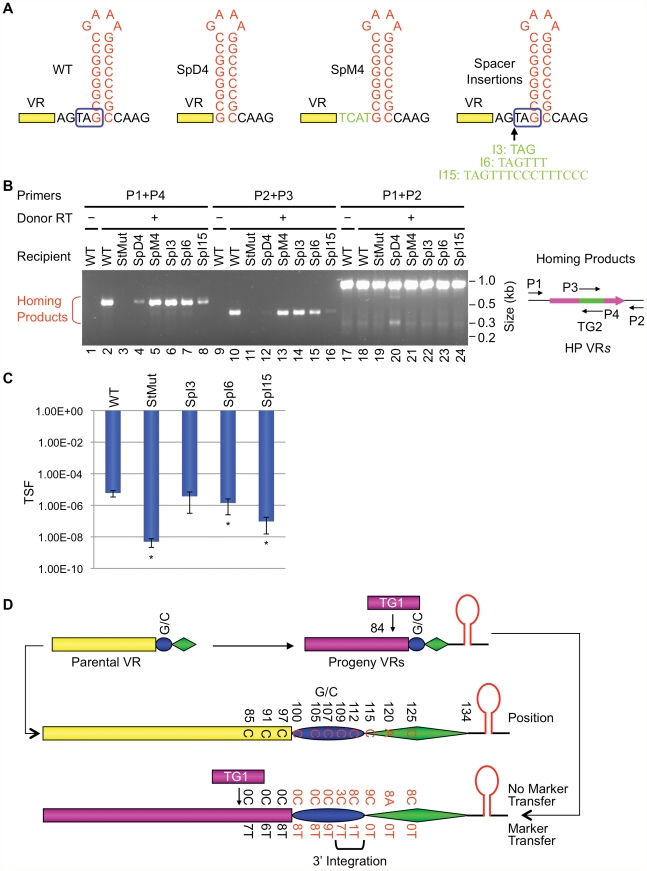
The VR-hairpin/cruciform spacer affects target recognition efficiency but not 3′ cDNA integration site. (A) Modifications introduced in the spacer between VR and the hairpin/cruciform structure. SpD4, the 4 bp spacer was deleted; SpM4, residues of the 4 bp spacer were switched to their complementary nucleotides; SpI3, SpI6 and SpI15 are spacer insertion mutants that retain the *mtd* stop codon for proper Mtd production. Boxed TAG, *mtd* stop codon. (B) Effects of spacer modifications on DGR mutagenic homing. PCR-homing assays are shown along with primers used. (C) Effects of spacer modifications on phage tropism switching. The bars represent mean TSF ± s.d. P values comparing mutants to WT in Student's t test are indicated with asterisks. *P<0.05. Tropism switching data are not available for constructs SpD4 and SpM4, as both modifications eliminate the *mtd* stop codon and do not produce functional phage particles. (D) Summary of marker coconversion analysis with BPP-1Δ*ATR*SpI6. BPP-1Δ*ATR*SpI6 phage particles were used for single-cycle lytic infection of RB50 cells transformed with marked donor plasmids. Progeny phage DNAs were used for PCR-based DGR homing assays. PCR products were cloned and sequenced and marker coconversion data are summarized. Nucleotide residues in the parental VR that correspond to the marked positions in TR are shown in the center. Numbers of progeny VRs with or without transferred markers at designated sites are shown at the bottom. The deduced cDNA integration region at the 3′ end of VR is indicated by a bracket.

In the experiments in Figure 8, we tested the effects of altering the position of the hairpin/cruciform with respect to the 3′ boundary of VR and probed sequence requirements for the intervening region. SpM4 (Figure 8A), in which the 4 residues in the spacer were switched to the complementary nucleotides, retained WT activity (Figure 8B). In contrast, deletion of the spacer (SpD4) resulted in a significant decrease in target function. The SpM4 and SpD4 mutations eliminate the *mtd* stop codon and generate non-infective phages, obviating the ability to measure tropism switching. Nonetheless, their relative levels of activity were readily apparent in PCR-homing assays. Expansion of the spacer was tolerated to a greater extent than deletion. SpI3, which has a 3 bp insertion in the spacer (Figure 8A), showed no significant defect in PCR-homing or phage tropism switching assays (Figure 8B and 8C), but longer insertions gradually decreased target site function.

The SpI6 insertion, which increases the distance between the hairpin/cruciform structure and the 3′ end of VR by 6 bp, retains a measurable level of activity. We took advantage of this and used a marker coconversion assay (Figure S8; [4]) to determine the relationship between the position of the hairpin/cruciform structure and the site at which information transfer initiates. As summarized in Figure 8D, our coconversion assay measured transfer of nucleotide polymorphisms from tagged TR donors to a recipient VR carrying the SpI6 mutation using PCR-based homing assays (data not shown). With the WT recipient, a coconversion boundary occurs between positions 107 and 112, and this was interpreted as representing the site at which TR-derived cDNA synthesis initiates [4]. As shown in Figure 8D, the coconversion boundary remains essentially unchanged in the SpI6 mutant. Although the position of the hairpin structure affects the efficiency of DGR homing, it does not determine the site at which cDNA is integrated at the 3′ end of VR.

### Engineering the BPP-1 DGR to target a heterologous gene

To determine if the results presented here complete our understanding of DGR-encoded requirements for retrohoming to a target gene, we applied them as engineering principles in an attempt to construct a functional, synthetic, TR/VR system. For a DNA sequence to serve as a recipient VR, three conditions must be met. First, it must be adjacent to an IMH region with functional (GC)_14_ and 21 bp elements at its 3′ end [1], [4]. Second, the IMH region must be followed by inverted repeats capable of forming a hairpin/cruciform structure of appropriate size, composition and distance from IMH. And finally, sufficient VR/TR sequence homology must be provided to allow efficient upstream (5′) cDNA integration. In recent studies we have shown that although short stretches of nucleotide identity (≥8 bp) between the TR-derived cDNA and VR target sequences are sufficient to complete the homing reaction, homing efficiency is increased with longer (≥19 bp) stretches of homology [4]. With these parameters in mind, we tested our ability to engineer the BPP-1 DGR to target a heterologous reporter gene (*aph3′Ia*; [15]) which provides facile detection of targeting events by antibiotic selection.

**Figure 9:**
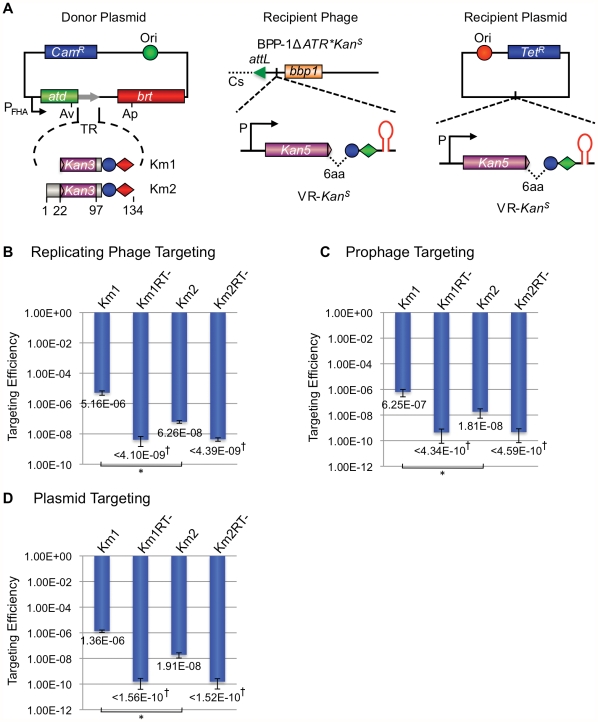
The BPP-1 DGR can be engineered to target a kanamycin-resistance gene. (A) Donor and recipient constructs used for *Kan^R^* gene targeting. The left panel shows two donor plasmids, pMX-Km1 and pMX-Km2, both with engineered TRs containing the last 36 bp of the *Kan^R^* ORF. The *Kan^R^* reporter gene on recipient VRs has a truncation of the last 6 codons and is placed upstream of the IMH and hairpin/cruciform elements. The recipient cassette was inserted between *attL* and *bbp1* of phage BPP-1ΔATR* (Recipient Phage, middle) or in a pMMB208-derived plasmid (Recipient Plasmid, left). (B) Engineered BPP-1 DGRs can target a *Kan^R^* reporter gene on a replicating phage. Targeting assays were carried out by phage BPP-1ΔATR*Kan^S^ lytic infection of RB50 cells carrying the indicated donor plasmids. Targeting efficiencies were determined as the relative numbers of Kan^R^ cells in lysogens generated with fresh RB50 cells and the progeny phages. The bars for Km1 and Km2 represent mean ± s.d. *P<0.01 in a Student's t test. (C) Engineered BPP-1 DGRs can target a *Kan^R^* reporter gene on a prophage in the bacterial chromosome. BPP-1ΔATR*Kan^S^ lysogens transformed with indicated donor plasmids were used for targeting assays. Resulting cells were directly plated on selective (+Kan) or non-selective plates to determine relative numbers of Kan^R^ cells. The bars represent mean ± s.d. *P<0.05 in a Student's t test. (D) Engineered BPP-1 DGRs can target a *Kan^R^* reporter gene on a plasmid. Following induction for donor plasmid expression and *Kan^R^* gene targeting, RB50 cells transformed with both donor and recipient plasmids were plated on selective (+Kan) or non-selective plates to determine targeting efficiencies as in (C). The bars represent mean ± s.d. *P<0.002 in a Student's t test. **^†^**No Kan^R^ colonies were observed for RT-deficient donors and the numbers represent limits of detection.

The recipient VR-*Kan^S^* cassette shown in Figure 9A contains an *aph3′Ia* kanamycin resistance (*Kan^R^*) allele with a 3′ deletion that renders it nonfunctional by removing coding sequences for 6 essential C-terminal residues. The truncated gene was placed immediately upstream of IMH, followed by the hairpin/cruciform-forming inverted repeats from the BPP-1 DGR. Transcription is directed by the native *aph3′Ia* promoter. The donor plasmid expresses *avd*, *brt*, and one of two engineered TRs (TR-Km1, TR-Km2) from the P_fha_ promoter. Both TRs contain the intact 3′ end of the *aph3′Ia* open reading frame, followed by two consecutive stop codons and sequences 97–134 from the 3′ end of the BPP-1 TR. For TR-Km2, the *aph3′Ia* fragment is also flanked, at its 5′ end, by the first 22 residues of the BPP-1 TR. DGR-mediated retrotransposition from the donor TR constructs to the VR-*Kan^S^* recipient should regenerate a full-length *aph3′Ia* gene conferring *Kan^R^*.

We first tested whether targeting can occur in the context of a replicating phage. BPP-1ΔATR*Kan^S^ carries the VR-*Kan^S^* cassette inserted between *attL* and *bbp1* on the left arm of the prophage genome [16], along with a deletion of *avd*, TR and *brt* and a series of synonymous substitutions in IMH to inactivate the *mtd* VR (Figure 7A). *B. bronchiseptica* RB50 carrying the TR-Km1 or -Km2 donor plasmid, or derivatives with a null mutation in *brt*, were infected with BPP-1ΔATR*Kan^S^ and targeting efficiencies were determined by infecting RB50 with progeny phages and measuring relative numbers of Kan^R^ lysogens. Kan^R^ lysogens were readily detected when targeting occurred from Brt+ TR donors, but not Brt− donors (Figure 9B), and sequence analysis showed that Kan^R^ resulted from the regeneration of full-length *aph3′Ia* alleles which often contained mutations at positions corresponding to adenines in donor TRs (Figures S9 and S10). It is interesting to note that the TR-Km1 donor was significantly more efficient than TR-Km2. This suggests that the majority of cDNAs are extended to the 5′ termini of these short synthetic TRs, and target (VR) homology to the extreme 3′ ends of the extension products may be advantageous for cDNA integration.

We also tested the ability to target the VR-*Kan^S^* cassette when present on a resident prophage in the bacterial chromosome or on a plasmid. In the experiment in Figure 9C, RB50/BPP-1ΔATR*Kan^S^ lysogens were transformed with donor plasmids under conditions that suppress P_fha_ promoter activity. Following a 6 hr pulse of P_fha_ induction, cells were plated under promoter-suppressing conditions on media with or without kanamycin. In Figure 9D, a similar protocol was used to target a VR-*Kan^S^* cassette carried on a medium copy number plasmid in RB50 cells containing a TR donor plasmid, but no other phage sequences. In both experiments, Kan^R^ colonies were readily detected when targeting occurred from Brt+, but not Brt− TR donors, and sequence analysis showed characteristic patterns of adenine mutagenesis (Figures S11, S12, S13, S14). Taken together, our results demonstrate the ability to engineer a VR/TR system that targets a heterologous reporter gene on a phage, plasmid or bacterial genome. The data in Figure 9D show that no BPP-1 phage products, other than those encoded in the DGR, are required for mutagenic retrohoming.

## Discussion

Understanding DGR target site recognition requires a precise definition of *cis*-acting sequences important for retrohoming. Our analysis of the boundaries of the BPP-1 DGR target showed that sequences upstream of VR are dispensable, as predicted by previous results [4]. More importantly, we show that homing is facilitated by an element downstream of VR, beyond the point at which TR/VR homology ends. Sequence analysis, mutagenesis, and structure-specific nuclease assays demonstrated that GC-rich inverted repeats directly following VR form a hairpin/cruciform structure that plays a critical role in retrohoming. Highly similar elements are present in analogous locations in many phage- or prophage-related DGRs (Figure 5), and hairpin/cruciform structures are predicted for the majority of DGRs that naturally reside on bacterial chromosomes and plasmids as well [Gingery et al., unpublished data]. We propose that DNA hairpin formation near the 3′ end of VR is a conserved requirement for DGR-mediated retrohoming.

For the BPP-1 DGR target, the 8 bp stem appears to function as a structure that is dependent on nucleotide composition but not sequence. In contrast, the loop of the hairpin/cruciform structure is constrained in size and sequence and conforms to the consensus, 5′-GRNA, derived from comparisons with other phage-related DGRs. This suggests that loop sequence and size may be important for stabilizing the hairpin/cruciform structure [17], or for creating a strand bias in DNA cleavage by a host-encoded endonuclease. It is also possible that the loop is in direct physical contact with a critical component, such as Brt, Avd, a TR-containing RNA transcript, or other parts of the DGR target. By testing the effects of length and sequence variations between the hairpin/cruciform and VR, we found that distance is an important parameter, although some flexibility exists. Extending the spacer by 6 bp did not shift the marker coconversion boundary in the (GC)_14_ region during DGR homing [4], showing that the position of the hairpin/cruciform does not determine the site at which 3′ cDNA integration occurs.

DGRs are evolutionarily related to group II introns [1] and it is interesting to note that a subset of these retroelements, the group IIC introns, also target motifs with stem-loop structures [18]–[20]. In nature, group IIC introns are often found to be located short distances downstream of sequences encoding known or predicted factor-independent transcription terminators, which are composed of GC-rich stems with loops of varying sizes followed by poly-uridine stretches [18]–[20]. Using an *in vitro* mobility assay, Robart *et al.* [19] have shown that reconstituted ribonucleoprotein particles from the *Bacillus halodurans B.h.*I1 group IIC intron recognize structures in ssDNA that correspond to RNA hairpins formed during transcription termination. As observed with the BPP-1 DGR, the *B.h.*I1 mobility reaction was highly dependent on stem formation but not absolute sequence [19]. Stems shorter than 9 bp had significantly reduced activities in *in vitro* mobility assays, a longer stem (14 bp) retained function, and the efficiency of targeting correlated with GC content and predicted stem stability [19]. In contrast to our observations with the BPP-1 DGR, alterations in loop sequence had little effect on *B.h*.I1 mobility *in vitro* [19]. The adaptation of group IIC introns to recognize and insert downstream of factor-independent transcriptional terminators was proposed to provide a selective advantage by limiting their expression, avoiding the interruption of essential coding sequences, and facilitating horizontal spread as intrinisic terminators are common and conserved in bacteria [19]. For DGRs, we speculate that the ability to target sequences upstream of terminator-like stem-loop structures may have played a role in directing their sequence diversification capabilities to the 3′ coding regions of target genes.

The TPRT model for DGR homing postulates that cDNA synthesis initiates with a nick or double-strand break in the IMH (GC)_14_ sequence, providing a primer for reverse transcription of a TR-containing RNA transcript [4]. Analogous to target recognition by group IIC introns, the hairpin/cruciform structure may serve as a recognition element for a retrohoming complex that includes *trans*-acting DGR-encoded factors. A DNA endonuclease that might be responsible for cleavage awaits identification, and possibilities include Avd, Brt, a TR-derived catalytic RNA, or an unidentified host factor. It is also possible that the DNA hairpin/cruciform actively promotes single- or double-strand breaks. If DNA repair synthesis extends to the (GC)_14_ region, the elongating antisense strand could then be used for cDNA priming. DNA breaks at the hairpin/cruciform structure could be created by an endonuclease that cleaves the single-stranded loop, or by a structure-specific enzyme similar to T7 endonuclease I [21]. Since DNA cruciforms are structurally similar to Holiday junctions, host-encoded recombination proteins that function in resolving recombination intermediates could be involved [22]. The cDNA priming mechanism of the BPP-1 DGR appears to be different from that of mobile group II introns that lack a DNA endonuclease activity in their intron-encoded proteins [23]–[25]. Reverse transcription in retrohoming and ectopic transposition of these elements is proposed to be primed by either the leading or lagging strand during DNA replication, and strong strand-specific biases are observed [23]–[25]. Our observation that the BPP-1 DGR target sequence is orientation-independent suggests that DNA replication polarity does not play a significant role in cDNA priming. Although our results to date are consistent with TPRT, further studies are required to definitively characterize the mechanism of cDNA initiation and integration at the 3′ end of VR and to determine the precise role of the hairpin/cruciform structure in the retrohoming process.

The broad distribution of DGRs in nature attests to their utility, and prospects for adapting these elements for protein engineering applications are compelling. Our results demonstrate that the region containing the (GC)_14_ and 21 bp sequences in IMH, and an adjacent hairpin/cruciform, is sufficient to direct the DGR mutagenic homing machinery to a heterologous target gene through appropriate engineering of a cognate TR. Using similar design principles we have successfully targeted a tetracycline resistance determinant as well (HG and JFM, unpublished data). For DGRs to be useful tools, it will be necessary to engineer their activity to allow efficient and controlled diversification. Having defined the DGR-encoded *cis*- and *trans*-acting factors required to diversify heterologous sequences, efforts to optimize their activities can now proceed in an informed and comprehensive way. It will also be important to determine the effects of TR/VR size, composition, and position relative to *cis*-acting DGR elements, on the efficiency of diversifying heterologous sequences. In preliminary experiments, insertions of moderate size (up to ∼200 bp) at position 84 in the BPP-1 TR (134 bp) are transferred to VR and mutagenized at adenines, suggesting that sequences of >300 bp could be diversified by an engineered system (LVT, HG and JFM, unpublished data).

In addition to providing prodigious levels of diversity, mutagenic homing is a regenerative process that allows DGRs to operate through unlimited rounds to optimize variable protein functions [4]. This may be particularly advantageous for directed protein evolution since desired traits can be selected and continuously evolved in iterative cycles, without the need for library construction or other interventions, through a process that takes place entirely within bacterial cells.

## Materials and Methods

### Bacterial strains and phages

*B. bronchiseptica* strains RB50, RB53Cm, RB54 and ML6401 have been described [16]. The BPP-1Δ*ATR* lysogen was constructed from ML6401, an RB50 strain lysogenized with phage BPP-1, by deleting sequences from *avd* position 48 to position 882 of *brt*. Target region deletions/insertions and hairpin/cruciform modifications were introduced into the BPP-1Δ*ATR* lysogen through allelic exchange [1], [4] and are diagramed in the figures. The BPP-1ΔATR* lysogen contains multiple silent mutations at both the 5′ and 3′ ends of VR to inactivate it as a DGR target. It was used as the parental strain to create the BPP-1ΔATR*Kan^S^ lysogen, in which the *Kan^R^* gene *aph3′Ia* has sequences encoding the C-terminal 6 amino acid residues truncated and is placed upstream of IMH and the hairpin/cruciform structure as a reporter for heterologous gene targeting. The *aph3′Ia* allele also contains an AAA to CGC substitution resulting in K260R. The VR-*Kan^S^* reporter cassette was inserted between *attL* and *bbp1* of the phage genome. Phage BPP-1Δ*ATR* and its various derivatives were produced from the above lysogens.

### Plasmid constructs

Plasmid pMX-ΔTR23–96 has TR positions 23–96 deleted and replaced by a 30 bp PCR tag as in pMX-ΔTR23–84 [4]. Its RT-deficient derivative contains the YMDD to SMAA mutation at Brt positions 213–216 [3], [4]. Plasmids pMX1 and pMX1SMAA were used for phage tropism switching assays and have previously been described [4].

pUC-StWT is a pUC18-based plasmid containing the WT BPP-1 DGR target from position −6 upstream of VR to position +82 downstream of VR. pUC-StMut is its derivative with 7 residues in the 3′ half of the stem, proximal to the loop, mutated to their complementary nucleotides.

Plasmids pMX-TRC85T, pMX-TRC91T, pMX-TRC97T, pMX-TRC100T, pMX-TRC105T, pMX-TRC107T, pMX-TRC109T, pMX-TRC112T, pMX-TRC115T, pMX-TRC120T and pMX-TRC125T have been previously described [4].

Plasmids pMX-Km1 and pMX-Km2 were constructed from pMX-ΔTR23–96 for *Kan^R^* gene targeting, both containing the last 36 bp of *aph3′Ia*. The 36 bp sequence and its following two stop codons replace TR positions 1–96 in pMX-Km1 and TR positions 23–96 in pMX-Km2.

Plasmid pHGT-*Kan^S^* contains the VR-*Kan^S^* cassette described above and was used as the recipient plasmid for *Kan^R^* targeting. The plasmid also carries a tetracycline resistance gene.

### Phage production for DGR homing and tropism switching assays

Phage production for DGR functional assays was carried out by either single-cycle lytic infection or mitomycin C induction from lysogens as previously described [4], except for minor modifications as noted. For single-cycle lytic infection, *B. bronchiseptica* RB50 cells transformed with appropriate donor plasmids were grown overnight at 37°C in Luria-Bertani (LB) media containing 25 µg/ml of chloramphenicol (Cam), 20 µg/ml streptomycin (Str), and 10 mM nicotinic acid to modulate to the Bvg^−^ phase and prevent transcription from the P_fha_ promoter. An amount of cells equal to 1 ml of culture (OD_600_ = 1.0) was pelleted, rinsed, and resuspended in 2.5 ml Stainer Scholte (SS) medium [26] containing 25 µg/ml Cam and 20 µg/ml Str (SS+Cam+Str). Cultures were grown for 3 hr at 37°C to modulate bacteria to the Bvg^+^ phase and activate P_fha_ promoter expression. An aliquot of 500 µl from each culture was used for OD_600_ measurement and cell number calculation. Phage particles were added to the rest of the culture at a multiplicity of infection of ∼2.0. Following 1 hr incubation at 37°C for phage absorption, infected cells were pelleted and resuspended in 1 ml of fresh, prewarmed SS+Cam+Str media and incubated at 37°C for 3 hr post phage addition to allow completion of a single cycle of phage development. Progeny phages were harvested following chloroform extraction.

For phage production from lysogens, RB50 derivatives carrying appropriate prophages and donor plasmids were grown and modulated to the Bvg^+^ phase as in single-cycle lytic infections. Phage production was induced with 0.2 µg/ml mitomycin C for 3 hr at 37°C. Progeny phages were harvested by chloroform extraction.

### BPP-1 phage tropism switching and PCR-based DGR homing assays

Phage tropism switching and DGR homing assays have been previously described [4].

### Analysis of hairpin/cruciform formation in plasmid DNA *in vitro*

Plasmids containing the WT BPP-1 DGR target and the StMut mutation were isolated from *E. coli* DH5αλpir cells using the QIAprep Spin miniprep kit (Qiagen). Plasmids were linearized by digestion with *Bgl*I as indicated. To analyze hairpin/cruciform structure formation in supercoiled or relaxed DNAs, 0.5 µg of supercoiled or linearized plasmids were treated with 10 units of T7 DNA endonuclease I (New England Biolabs, Ipswich, MA) for 40 minutes as in Miller *et al.* [11]. The reactions were terminated by phenol-chloroform-isoamyl alcohol (25∶24∶1) extraction and DNAs were precipitated with ethanol. T7 DNA endonuclease I cleavage sites were determined by primer extension with 5′-end ^32^P-labeled primers using Vent (exo-) DNA polymerase (New England Biolabs, Ipswich, MA) as in Miller *et al.* [11], except that 5% DMSO was added for GC-rich templates. Primer extension products were resolved on 6% polyacrylamide/8 M urea gels, alongside Sanger sequencing ladders generated with the same labeled primers and a plasmid template containing the WT target.

### Targeting of a *Kan^R^* gene by engineered BPP-1 phage DGRs

To target the *Kan^R^* gene on a replicating phage, BPP-1ΔATR*Kan^S^ phage particles were used for single-cycle lytic infection of RB50 cells transformed with appropriate donor plasmids, similar to phage production by single-cycle lytic infection described above. Progeny phages were titered and ∼10^11^ pfu of different phages were added to 25 ml RB50 cells (OD_600_ = 1.2) in SS+Str media for 8.0 hr to reestablish lysogens. Cells were pelleted and resuspended in 5 ml LB and serial dilutions were plated on LB+NA+Str and LB+NA+Str+Kan (50 µg/ml) to determine *Kan^R^* gene targeting frequencies. Lysogen reestablishment efficiencies ranged from 60% to 100% based on PCR analysis of 10 colonies each picked on LB+NA+Str plates using phage specific primers. *Kan^R^* targeting efficiency for each donor plasmid was determined as the ratio of colony forming units (cfu) on LB+NA+Str+Kan plates to those on LB+NA+Str, calibrated with the lysogen reestablishment efficiency for that sample.

To target the *Kan^R^* gene on a prophage in the bacterial chromosome, RB50 cells lysogenized with phage BPP-1ΔATR*Kan^S^ were transformed with appropriate donor plasmids. Starting cultures were grown overnight in LB+NA+Str+Cam as described above. An amount of cells equal to 1 ml of culture (OD_600_ = 1.0) was pelleted, rinsed, and resuspended in 2.5 ml SS+Cam+Str and grown at 37°C for 6 hours. Serial dilutions were plated on LB+NA+Str and LB+NA+Str+Kan (50 µg/ml) to determine *Kan^R^* gene targeting frequencies. *Kan^R^* targeting efficiencies were determined as relative numbers of *Kan^R^* cells as above. To target the *Kan^R^* gene on a plasmid, the recipient plasmid pHGT-*Kan^S^* and appropriate donors were transformed into RB50 cells and analyzed similarly. Tetracycline was added to 5.0 µg/ml for recipient plasmid maintenance.

## Supporting Information

Figure S1 Alignment of BPP-1 DGR target deletion constructs showing deletion boundary sequences. (A) Alignment of 5′ deletion constructs (Figure 1C) with the corresponding region of the WT sequence. The WT sequence extends from position −10 upstream of VR to VR position 134 (last nucleotide). The 5′ end of VR and the (GC)_14_ element for the WT sequence are marked. Sequences that replace the VR deletions in 5′Δ133 and 5′Δ153 are underlined in blue. The “inserted” sequences are significantly different from the original ones, although 5′Δ133 regains a C residue at position −1. (B) Alignment of 3′ deletion constructs (Figure 1C) with the corresponding region of the WT sequence. The WT sequence extends from the 5′ end of VR to the second codon of *avd*. The (GC)_14_ element and the potential hairpin region for the WT sequence are marked. Sequences that replace the deletions in 3′Δ47, 3′Δ68, 3′Δ82 and 3′Δ103 are underlined in blue. The “inserted” sequences are significantly different from the original ones. Sequences downstream of the potential hairpin structure in 3′Δ54 and 3′Δ58 are shown in Figure 1C and are not aligned here.
(PDF)

Figure S2 Alignment of homing products of recipient 5′Δ153 demonstrates cryptic 5′ cDNA integration and adenine mutagenesis. (A) PCR detection strategy for homing products of recipient 5′Δ153 and regions of the products aligned in (B) and (C). Primer annealing sites are indicated as small horizontal arrows. (B) Alignment of the homing products of recipient 5′Δ153 from VR position 21 to the end of the TG2 tag shows cryptic cDNA integration sites and adenine mutagenesis. The recipient has a 5′ deletion that includes the first 20 bp of VR. Cryptic integration sites are highlighted in pink. (C) Alignment of the transferred TG2 tag and its downstream VR sequence with the corresponding regions of the predicted homing product lacking adenine mutagenesis (TG2VR).
(PDF)

Figure S3 Analysis of homing products of recipient StRev. (A) PCR detection strategy for homing products of recipient StRev and regions of the products aligned in (B) and (C). Primer annealing sites are indicated as small horizontal arrows. (B) Alignment of the homing products of recipient StRev from the first position of VR to the end of the TG2 tag with the corresponding region of the predicted homing product lacking adenine mutagenesis (VR5′end). Adenine mutagenesis is observed in 8/15 cloned homing products. (C) Alignment of homing products of recipient StRev from the beginning of TG2 to the start codon of *avd* with the corresponding regions of the predicted WT homing product lacking adenine mutagenesis (wtHP3′end). The hairpin region is underlined in red to show complementary changes. Adenine mutagenesis is observed in 5/20 cloned homing products.
(PDF)

Figure S4 Sequence analysis of tropism switching products of phage BPP-1Δ*ATR* with WT hairpin/cruciform structures. Sequences from the beginning of VR to the start codon of *avd* of five progeny phages with switched tropisms were aligned with the corresponding region of the predicted WT homing product lacking adenine mutagenesis (TR99VR). The hairpin region is underlined in red and adenine mutagenesis is observed in all five progeny phages with switched tropisms.
(PDF)

Figure S5 Sequence analysis of tropism switching products of phage BPP-1Δ*ATR* StMut. Sequences from the beginning of VR to the start codon of *avd* of five tropism-switched progeny phages of recipient StMut were aligned with the corresponding region of the predicted WT homing product lacking adenine mutagenesis (TR99VR). The hairpin region is underlined in red to show disruption of the structure. Adenine mutagenesis is observed in all five phage tropism switching products.
(PDF)

Figure S6 Sequence analysis of tropism switching products of phage BPP-1Δ*ATR* StRev. Sequences from the beginning of VR to the start codon of *avd* of five tropism-switched progeny phages of recipient StRev were aligned with the corresponding region of the predicted WT homing product lacking adenine mutagenesis (TR99VR). The hairpin region is underlined in red to show complementary changes. Adenine mutagenesis is observed in all five phage tropism switching products.
(PDF)

Figure S7 Analysis of homing products of recipient VRInv. (A) PCR detection strategy for homing products of recipient VRInv and regions of the products aligned in (B) and (C). Primer annealing sites are indicated as small horizontal arrows. (B) Alignment of homing products of recipient VRInv from the 5′ end of VR to the end of the TG2 tag with the corresponding region of the predicted homing product lacking adenine mutagenesis (VR5′end). Adenine mutagenesis is observed in 5/10 cloned homing products. (C) Alignment of homing products of recipient VRInv from the beginning of TG2 to the end of VR with the corresponding region of the predicted WT homing product lacking adenine mutagenesis (VR3′end). Adenine mutagenesis is observed in 2/9 cloned homing products.
(PDF)

Figure S8 Outline of marker coconversion assay with recipient phage BPP-1Δ*ATR*SpI6. (A) PCR-based DGR homing assays with marked donor plasmids. Markers were introduced into plasmid pMX-TG1cAA [4]. The TR contains a 36 bp insert (TG1) at position 84. Grey and pink arrows represent TR and progeny VRs, respectively. Small horizontal arrows indicate primers used for homing assays: P7 and P8 are sense- and antisense-strand primers annealing upstream and downstream of VR, respectively; P9 and P10 (Table 1) are sense- and antisense-strand primers, respectively, that anneal to TG1. *Cam^R^*, chloramphenicol resistance gene. (B) Schematic of coconversion experiments to determine 3′ marker transfer boundaries. Single C to T markers downstream of the TG1 tag in donor TRs are indicated and the constructs have been previously described [4]. Markers (red T residues) are transferred to VR only if they are located between the TR positions corresponding to 3′ and 5′ cDNA integration sites in VR.
(PDF)

Figure S9 Sequence analysis of replicating phage *Kan^R^* targeting products with the pMX-Km1 donor. Sequences from the beginning of VR-*Kan^S^* to the end of the hairpin structure were aligned with the corresponding region of the predicted *Kan^R^* targeting product lacking adenine mutagenesis (KmHP). The targeting assay was carried out with BPP-1ΔATR*Kan^S^ single-cycle lytic infection of RB50 cells transformed with donor plasmid pMX-Km1. Progeny phages were used to generate lysogens in RB50 cells, which were analyzed on plates with and without kanamycin to determine the efficiency of *Kan^R^* targeting. *Kan^R^* clones were sequenced to verify regeneration of full-length *Kan^R^* genes. Adenine mutagenesis is observed in 7/10 clones.
(PDF)

Figure S10 Sequence analysis of replicating phage *Kan^R^* targeting products with the pMX-Km2 donor. Sequences from the beginning of VR-*Kan^S^* to the end of the hairpin structure were aligned with the corresponding region of the predicted *Kan^R^* targeting product lacking adenine mutagenesis (KmHP). The targeting assay was carried out with BPP-1ΔATR*Kan^S^ single-cycle lytic infection of RB50 cells transformed with donor plasmid pMX-Km2. RB50 cells were lysogenized with progeny phages and subsequently analyzed on plates with and without kanamycin to determine the efficiency of *Kan^R^* targeting. *Kan^R^* clones were sequenced to verify regeneration of full-length *Kan^R^* genes. Adenine mutagenesis is observed in 7/11 clones.
(PDF)

Figure S11 Sequence analysis of prophage *Kan^R^* targeting products with the pMX-Km1 donor. Sequences from the beginning of VR-*Kan^S^* to the end of the hairpin structure were aligned with the corresponding region of the predicted *Kan^R^* retargeting product lacking adenine mutagenesis (KmHP). The targeting assay was carried out in BPP-1ΔATR*Kan^S^ lysogen cells transformed with donor plasmid pMX-Km1. Resulting cells were analyzed on plates with and without kanamycin to determine the efficiency of *Kan^R^* targeting. Kan^R^ clones were then sequenced to verify regeneration of full-length *Kan^R^* genes. Adenine mutagenesis is observed in 13/16 clones.
(PDF)

Figure S12 Sequence analysis of prophage *Kan^R^* targeting products with the pMX-Km2 donor. Sequences from the beginning of VR-*Kan^S^* to the end of the hairpin structure were aligned with the corresponding region of the predicted *Kan^R^* retargeting product lacking adenine mutagenesis (KmHP). The targeting assay was carried out in BPP-1ΔATR*Kan^S^ lysogen cells transformed with donor plasmid pMX-Km2. Resulting cells were plated on plates with and without kanamycin to determine the efficiency of *Kan^R^* targeting. Kan^R^ clones were sequenced to confirm regeneration of full-length *Kan^R^* genes. Adenine mutagenesis is observed in 13/16 clones.
(PDF)

Figure S13 Sequence analysis of plasmid *Kan^R^* targeting products with the pMX-Km1 donor. Sequences from the beginning of VR-*Kan^S^* to the end of the hairpin structure were aligned with the corresponding region of the predicted *Kan^R^* targeting product lacking adenine mutagenesis (KmHP). Targeting assay was carried out in RB50 cells transformed with both recipient plasmid pHGT-*Kan^S^* and donor plasmid pMX-Km1. Resulting cells were analyzed on plates with and without kanamycin to determine the efficiency of *Kan^R^* targeting. Kan^R^ clones were sequenced to verify regeneration of full-length *Kan^R^* genes. Adenine mutagenesis is observed in 6/7 clones.
(PDF)

Figure S14 Sequence analysis of plasmid *Kan^R^* targeting products with the pMX-Km2 donor. Sequences from the beginning of VR-*Kan^S^* to the end of the hairpin structure were aligned with the corresponding region of the predicted *Kan^R^* retargeting product lacking adenine mutagenesis (KmHP). The targeting assay was carried out in RB50 cells transformed with both recipient plasmid pHGT-*Kan^S^* and donor plasmid pMX-Km2. Resulting cells were plated on plates with and without kanamycin to determine the efficiency of *Kan^R^* targeting. Kan^R^ clones were then sequenced to confirm regeneration of full-length *Kan^R^* genes. Adenine mutagenesis is observed in 9/10 clones.
(PDF)
