# Supplementary material for: Target Site Recognition by a Diversity-Generating Retroelement
Source: PLoS Genet. 2011 Dec 15;7(12):e1002414. doi: 10.1371/journal.pgen.1002414 (PMC3240598; doi:10.1371/journal.pgen.1002414)
Supplement: Figure S1 — Alignment of BPP-1 DGR target deletion constructs showing deletion boundary sequences. (A) Alignment of 5′ deletion constructs (Figure 1C) with the corresponding region of the WT sequence. The WT sequence extends from position −10 upstream of VR to VR position 134 (last nucleotide). The 5′ end of VR and the (GC)14 element for the WT sequence are marked. Sequences that replace the VR deletions in 5′Δ133 and 5′Δ153 are underlined in blue. The “inserted” sequences are significantly different from the original ones, although 5′Δ133 regains a C residue at position −1. (B) Alignment of 3′ deletion constructs (Figure 1C) with the corresponding region of the WT sequence. The WT sequence extends from the 5′ end of VR to the second codon of avd. The (GC)14 element and the potential hairpin region for the WT sequence are marked. Sequences that replace the deletions in 3′Δ47, 3′Δ68, 3′Δ82 and 3′Δ103 are underlined in blue. The “inserted” sequences are significantly different from the original ones. Sequences downstream of the potential hairpin structure in 3′Δ54 and 3′Δ58 are shown in Figure 1C and are not aligned here. (PDF) [file pgen.1002414.s001.pdf]

A

```
                           VR
WT5'      ACGCCCAGCCCGCTGCTGCGCTATTCGGCGGCGCCTGGAACGGCACGTCGCTCTCGGGTT 60
5'Δ105    ACGCCCAGCCCGCTGCTGCGCTATTCGGCGGCGCCTGGAACGGCACGTCGCTCTCGGGTT 60
5'Δ133    GAACATGTTCCGCTGCTGCGCTATTCGGCGGCGCCTGGAACGGCACGTCGCTCTCGGGTT 60
5'Δ153    CGCCACGAGCGCGTGGAACAGAACATGTTCGCGCCTGGAACGGCACGTCGCTCTCGGGTT 60
             *     *   **   *   *   *   ******************************

                                                             G/C
WT        CTCGCGCTGCGCTCTGGTACAGCGGGCCGTCGTTCTCGTTCGCGTTCTTCGGGGCGCGCG 120
5'Δ105    CTCGCGCTGCGCTCTGGTACAGCGGGCCGTCGTTCTCGTTCGCGTTCTTCGGGGCGCGCG 120
5'Δ133    CTCGCGCTGCGCTCTGGTACAGCGGGCCGTCGTTCTCGTTCGCGTTCTTCGGGGCGCGCG 120
5'Δ153    CTCGCGCTGCGCTCTGGTACAGCGGGCCGTCGTTCTCGTTCGCGTTCTTCGGGGCGCGCG 120
          ************************************************************

WT        GCGTCTGTGACCACCTGATTCTTG 144
5'Δ105    GCGTCTGTGACCACCTGATTCTTG 144
5'Δ133    GCGTCTGTGACCACCTGATTCTTG 144
5'Δ153    GCGTCTGTGACCACCTGATTCTTG 144
          ************************
```

B

```
WT3'      CGCTGCTGCGCTATTCGGCGGCGCCTGGAACGGCACGTCGCTCTCGGGTTCTCGCGCTGC 60
3'Δ47     CGCTGCTGCGCTATTCGGCGGCGCCTGGAACGGCACGTCGCTCTCGGGTTCTCGCGCTGC 60
3'Δ68     CGCTGCTGCGCTATTCGGCGGCGCCTGGAACGGCACGTCGCTCTCGGGTTCTCGCGCTGC 60
3'Δ82     CGCTGCTGCGCTATTCGGCGGCGCCTGGAACGGCACGTCGCTCTCGGGTTCTCGCGCTGC 60
3'Δ103    CGCTGCTGCGCTATTCGGCGGCGCCTGGAACGGCACGTCGCTCTCGGGTTCTCGCGCTGC 60
          ************************************************************

                                                     G/C
WT3'      GCTCTGGTACAGCGGGCCGTCGTTCTCGTTCGCGTTCTTCGGGGCGCGCGGCGTCTGTGA 120
3'Δ47     GCTCTGGTACAGCGGGCCGTCGTTCTCGTTCGCGTTCTTCGGGGCGCGCGGCGTCTGTGA 120
3'Δ68     GCTCTGGTACAGCGGGCCGTCGTTCTCGTTCGCGTTCTTCGGGGCGCGCGGCGTCTGTGA 120
3'Δ82     GCTCTGGTACAGCGGGCCGTCGTTCTCGTTCGCGTTCTTCGGGGCGCGCGGCGTCTGTGA 120
3'Δ103    GCTCTGGTACAGCGGGCCGTCGTTCTCGTTCGCGTTCTTCGGGGCGCGCGGCGCTGCAGC 120
          *****************************************************     *

                                 Hairpin
WT3'      CCACCTGATTCTTGAGTAGCGGGGCCGAAAGGCCCCGCCAAGGCAACCGATGGAA 175
3'Δ47     CCACCTGATTCTTGAGTAGCGGGGCCGAAAGGCCCCGCCAAGGCAACCGCTGCAG 175
3'Δ68     CCACCTGATTCTTGAGTAGCGGGGCCGACTGCAGCGGTTTCTCGCCTCGTGGTCG 175
3'Δ82     CCACCTGATTCTTGCTGCAGCGGTTTCTCGCCTCGTGGTCGGGTCATGCGCAATG 175
3'Δ103    GGTTTCTCGCCTCGTGGTCGGGTCATGCGCAATGGGCCGACACCCACAACCTTTT 175
                    ** *       *
```
