# Supplementary material for: Target Site Recognition by a Diversity-Generating Retroelement
Source: PLoS Genet. 2011 Dec 15;7(12):e1002414. doi: 10.1371/journal.pgen.1002414 (PMC3240598; doi:10.1371/journal.pgen.1002414)
Supplement: Figure S2 — Alignment of homing products of recipient 5′Δ153 demonstrates cryptic 5′ cDNA integration and adenine mutagenesis. (A) PCR detection strategy for homing products of recipient 5′Δ153 and regions of the products aligned in (B) and (C). Primer annealing sites are indicated as small horizontal arrows. (B) Alignment of the homing products of recipient 5′Δ153 from VR position 21 to the end of the TG2 tag shows cryptic cDNA integration sites and adenine mutagenesis. The recipient has a 5′ deletion that includes the first 20 bp of VR. Cryptic integration sites are highlighted in pink. (C) Alignment of the transferred TG2 tag and its downstream VR sequence with the corresponding regions of the predicted homing product lacking adenine mutagenesis (TG2VR). (PDF) [file pgen.1002414.s002.pdf]

A

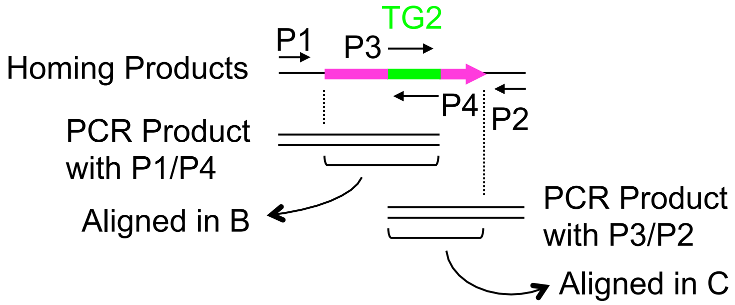

B

```
B4   GCGCCTGGAACGGCACGTCGCTCTCGGGTTCTCGCGCTGCGCTATTCGGCGGCGGGTCTG 60
D4   GCGCCTGGAACGGCACGTCGCTCTCGGGTTCTCGCGCTGCGCTATTCGGCGGCAGATCTG 60
E4   GCGCCTGGAACGGCACGTCGCTCTCGGGTTCTCGCGCTGCGCGATTCGGCGGCAGATCTG 60
F4   GCGCCTGGAACGGCACGTCGCTCTCGGGTTCTCGCGCTGCGCTATTCGGCGGCAGATCTG 60
G4   GCGCCTGGAACGGCACGTCGCTCTCGGGTTCTCGCGCTGCGCTATTCGGCGGCAGGTCTG 60
H4   GCGCCTGGAACGGCACGTCGCTCTCGGGTTCTCGCGCTGCGCTATTCGGCGGCAGGTCTG 60
     ****************************************** ********** * ****
```

Parental VR-derived

TR-derived

```
B4   TCTCCGTTTGTGTTCCTGTGCTAAGCTT 88
D4   TCTGCGTTTGTGTTCCTGTGCTAAGCTT 88
E4   TCTGCGTTTGTGTTCCTGTGCTAAGCTT 88
F4   TCTGCGTTTGTGTTCCTGTGCTAAGCTT 88
G4   TCTGCGTTTGTGTTCCTGTGCTAAGCTT 88
H4   TCTGCGTTTGTGTTCCTGTGCTAAGCTT 88
     *** ************************
```

P4

```
A4   GCGCCTGGAACGGCACGTCGCTCTCGGGTTCTCGCGCTGCGCTCTGTCTGCGTTTGTGTT
     CCTGTGCTAAGCTT 74
```

Parental VR-derived

TR-derived

P4

```
C4   GCGCCTGGAACGGCACGTCGCTCTCGGGTTCTCGCGCTTCGGCGGCGGTTCTGTCTGCGT
     TTGTGTTCCTGTGCTAAGCTT 81
```

Parental VR-derived

TR-derived

P4

C

```
TG2VR    ---AGATCTGTCTGCGTTTGTGTTCCTGTGCTAGCCATCGGGGCGCGCGGCGTCTGTGAC 57
A9       TCTAGATCTGTCTGCGTTTGTGTTCCTGTGCGACCCATCGGGGCGCGCGGCGTCTGTGGC 60
B9       TCTAGATCTGTCTGCGTTTGTGTTCCTGTGCTAGCCATCGGGGCGCGCGGCGTCTGTGAC 60
C9       TCTAGATCTGTCTGCGTTTGTGTTCCTGTGCTAGCCTTCGGGGCGCGCGGCGTCTGTGAC 60
D9       TCTAGATCTGTCTGCGTTTGTGTTCCTGTGCTAGCCATCGGGGCGCGCGGCGTCTGTGAC 60
E9       TCTAGATCTGTCTGCGTTTGTGTTCCTGTGCTAGCCATCGGGGCGCGCGGCGTCTGTGAC 60
F9       TCTAGATCTGTCTGCGTTTGTGTTCCTGTGTTAGCCATCGGGGCGCGCGGCGTCTGTGAC 60
G9       TCTAGATCTGTCTGCGTTTGTGTTCCTGTGCTAGCCATCGGGGCGCGCGGCGTCTGTGAC 60
H9       TCTAGATCTGTCTGCGTTTGTGTTCCTGTGCTAGCCGTCGGGGCGCGCGGCGTCTGTGAC 60
            ***************************  * ** ********************* *
                       P3                               G/C

TG2VR    CACCTGATTCTTG 70
A9       CACCTGATTCTTG 73
B9       CACCTGATTCTTG 73
C9       CACCTGATTCTTG 73
D9       CACCTGATTCTTG 73
E9       CACCTGATTCTTG 73
F9       CACCTGATTCTTG 73
G9       CACCTGATTCTTG 73
H9       CACCTGATTCTTG 73
         *************
```
