# Supplementary material for: Target Site Recognition by a Diversity-Generating Retroelement
Source: PLoS Genet. 2011 Dec 15;7(12):e1002414. doi: 10.1371/journal.pgen.1002414 (PMC3240598; doi:10.1371/journal.pgen.1002414)
Supplement: Figure S3 — Analysis of homing products of recipient StRev. (A) PCR detection strategy for homing products of recipient StRev and regions of the products aligned in (B) and (C). Primer annealing sites are indicated as small horizontal arrows. (B) Alignment of the homing products of recipient StRev from the first position of VR to the end of the TG2 tag with the corresponding region of the predicted homing product lacking adenine mutagenesis (VR5′end). Adenine mutagenesis is observed in 8/15 cloned homing products. (C) Alignment of homing products of recipient StRev from the beginning of TG2 to the start codon of avd with the corresponding regions of the predicted WT homing product lacking adenine mutagenesis (wtHP3′end). The hairpin region is underlined in red to show complementary changes. Adenine mutagenesis is observed in 5/20 cloned homing products. (PDF) [file pgen.1002414.s003.pdf]

A

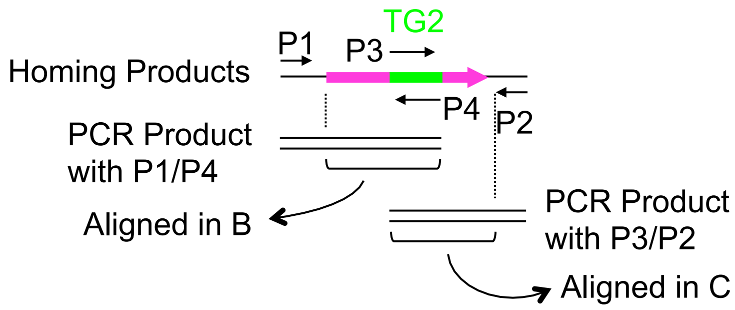

B

```
VR5'end     CGCTGCTGCGCTATTCGGCGGCAGATCTGTCTGCGTTTGTGTTCCTGTGCTAAGCTT 57
R2HP5'-01   CGCTGCTGCGCTATTCGGCGGCGTTTCTGTCTGCGTTTGTGTTCCTGTGCTAAGCTT 57
R2HP5'-02   CGCTGCTGCGCTATTCGGCGGCAGGTCTGTCTGCGTTTGTGTTCCTGTGCTAAGCTT 57
R2HP5'-03   CGCTGCTGCGCTATTCGGCGGCGGATCTGTCTGCGTTTGTGTTCCTGTGCTAAGCTT 57
R2HP5'-04   CGCTGCTGCGCTATTCGGCGGCAGATCTGTCTGCGTTTGTGTTCCTGTGCTAAGCTT 57
R2HP5'-05   CGCTGCTGCGCTATTCGGCGGCAGATCTGTCTGCGTTTGTGTTCCTGTGCTAAGCTT 57
R2HP5'-06   CGCTGCTGCGCTATTCGGCGGCAGATCTGTCTGCGTTTGTGTTCCTGTGCTAAGCTT 57
R2HP5'-07   CGCTGCTGCGCTATTCGGCGGCGGATCTGTCTGCGTTTGTGTTCCTGTGCTAAGCTT 57
R2HP5'-08   CGCTGCTGCGCTATTCGGCGGCAGATCTGTCTGCGTTTGTGTTCCTGTGCTAAGCTT 57
R2HP5'_09   CGCTGCTGCGCTATTCGGCGGCGGATCTGTCTGCGTTTGTGTTCCTGTGCTAAGCTT 57
R2HP5'_10   CGCTGCTGCGCTATTCGGCGGCAGGTCTGTCTGCGTTTGTGTTCCTGTGCTAAGCTT 57
R2HP5'_11   CGCTGCTGCGCTATTCGGCGGCAGATCTGTCTGCGTTTGTGTTCCTGTGCTAAGCTT 57
R2HP5'_12   CGCTGCTGCGCTATTCGGCGGCAGATCTGTCTGCGTTTGTGTTCCTGTGCTAAGCTT 57
R2HP5'_13   CGCTGCTGCGCTATTCGGCGGCAGCTCTGTCTGCGTTTGTGTTCCTGTGCTAAGCTT 57
R2HP5'_14   CGCTGCTGCGCTATTCGGCGGCAGATCTGTCTGCGTTTGTGTTCCTGTGCTAAGCTT 57
R2HP5'_15   CGCTGCTGCGCTATTCGGCGGCAGTTCTGTCTGCGTTTGTGTTCCTGTGCTAAGCTT 57
            **********************   ********************************
                                              P4
```

C

```
wtHP3'End   TCTAGATCTGTCTGCGTTTGTGTTCCTGTGCTAGCCATCGGGGCGCGCGGCGTCTGTGAC 60
R2HP3'_01   TCTAGATCTGTCTGCGTTTGTGTTTCTGTGCTAGCCATCGGGGCGCGCGGCGTCTGTGAC 60
R2HP3'_02   TCTAGATCTGTCTGCGTTTGTGTTCCTGTGCTAGCCATCGGGGCGCGCGGCGTCTGTGAC 60
R2HP3'_03   TCTAGATCTGTCTGCGTTTGTGTTCCTGTGCTAGCCATCGGGGCGCGCGGCGTCTGTGAC 60
R2HP3'_04   TCTAGATCTGTCTGCGTTTGTGTTCCTGTGCTAGCCATCGGGGCGCGCGGCGTCTGTGAC 60
R2HP3'_05   TCTAGATCTGTCTGCGTTTGTGTTCCTGTGCTAGCCATCGGGGCGCGCGGCGTCTGTGAC 60
R2HP3'_06   TCTAGATCTGTCTGCGTTTGTGTTCCTGTGCTAGCCATCGGGGCGCGCGGCGTCTGTGAC 60
R2HP3'_07   TCTAGATCTGTCTGCGTTTGTGTTCCTGTGCTAGCCATCGGGGCGCGCGGCGTCTGTGAC 60
R2HP3'_08   TCTAGATCTGTCTGCGTTTGTGTTCCTGTGCTAGCCATCGGGGCGCGCGGCGTCTGTGAC 60
R2HP3'_09   TCTAGATCTGTCTGCGTTTGTGTTCCTGTGCTAGCCATCGGGGCGCGCGGCGTCTGTGAC 60
R2HP3'_10   TCTAGATCTGTCTGCGTTTGTGTTCCTGTGCTAGCCTTCGGGGCGCGCGGCGTCTGTGAC 60
R2HP3'_11   TCTAGATCTGTCTGCGTTTGTGTTCCTGTGCTAGCCATCGGGGCGCGCGGCGTCTGTGAC 60
R2HP3'_12   TCTAGATCTGTCTGCGTTTGTGTTCCTGTGCTAGCCATCGGGGCGCGCGGCGTCTGTGAC 60
R2HP3'_13   TCTAGATCTGTCTGCGTTTGTGTTCCTGTGCTAGCCATCGGGGCGCGCGGCGTCTGTGAC 60
R2HP3'_14   TCTAGATCTGTCTGCGTTTGTGTTCCTGTGCTAGCCTTCGGGGCGCGCGGCGTCTGTGAC 60
R2HP3'_15   TCTAGATCTGTCTGCGTTTGTGTTCCTGTGCTGTCCGTCGGGGCGCGCGGCGTCTGTGAC 60
R2HP3'_16   TCTAGATCTGTCTGCGTTTGTGTTCCTGTGCTGTCCGTCGGGGCGCGCGGCGTCTGTGAC 60
R2HP3'_17   TCTAGATCTGTCTGCGTTTGTGTTCCTGTGCTAGCCATCGGGGCGCGCGGCGTCTGTGAC 60
R2HP3'_18   TCTAGATCTGTCTGCGTTTGTGTTCCTGTGCTAGCCATCGGGGCGCGCGGCGTCTGTGAC 60
R2HP3'_19   TCTAGATCTGTCTGCGTTTGTGTTCCTGTGCTAGCCATCGGGGCGCGCGGCGTCTGTGAC 60
R2HP3'_20   TCTAGATCTGTCTGCGTTTGTGTTCCTGTGCGTGCCATCGGGGCGCGCGGCGTCTGTGAC 60
            ************************ ******   ** ***********************
                           P3                             G/C

wtHP3'End   CACCTGATTCTTGAGTAGCGGGGCCGAAAGGCCCCGCCAAGGCAACCGATG 111
R2HP3'_01   CACCTGATTCTTGAGTAGGCCCCGGGAAACCGGGGCCCAAGGCAACCGATG 111
R2HP3'_02   CACCTGATTCTTGAGTAGGCCCCGGGAAACCGGGGCCCAAGGCAACCGATG 111
R2HP3'_03   CACCTGATTCTTGAGTAGGCCCCGGGAAACCGGGGCCCAAGGCAACCGATG 111
R2HP3'_04   CACCCGATTCTTGAGTAGGCCCCGGGAAACCGGGGCCCAAGGCAACCGATG 111
R2HP3'_05   CACCTGATTCTTGAGTAGGCCCCGGGAAACCGGGGCCCAAGGCAACCGATG 111
R2HP3'_06   CACCTGATTCTTGAGTAGGCCCCGGGAAACCGGGGCCCAAGGCAACCGATG 111
R2HP3'_07   CACCTGATTCTTGAGTAGGCCCCGGGAAACCGGGGCCCAAGGCAACCGATG 111
R2HP3'_08   CACCTGATTCTTGAGTAGGCCCCGGGAAACCGGGGCCCAAGGCAACCGATG 111
R2HP3'_09   CACCTGATTCTTGAGTAGGCCCCGGGAAACCGGGGCCCAAGGCAACCGATG 111
R2HP3'_10   CACCTGATTCTTGAGTAGGCCCCGGGAAACCGGGGCCCAAGGCAACCGATG 111
R2HP3'_11   CACCTGATTCTTGAGTAGGCCCCGGGAAACCGGGGCCCAAGGCAACCGATG 111
R2HP3'_12   CACCTGATTCTTGAGTAGGCCCCGGGAAACCGGGGCCCAAGGCAACCGATG 111
R2HP3'_13   CACCTGATTCTTGAGTAGGCCCCGGGAAACCGGGGCCCAAGGCAACCGATG 111
R2HP3'_14   CACCTGATTCTTGAGTAGGCCCCGGGAAACCGGGGCCCAAGGCAACCGATG 111
R2HP3'_15   CACCTGATTCTTGAGTAGGCCCCGGGAAACCGGGGCCCAAGGCAACCGATG 111
R2HP3'_16   CACCTGATTCTTGAGTAGGCCCCGGGAAACCGGGGCCCAAGGCAACCGATG 111
R2HP3'_17   CACCTGATTCTTGAGTAGGCCCCGGGAAACCGGGGCCCAAGGCAACCGATG 111
R2HP3'_18   CACCTGATTCTTGAGTAGGCCCCGGGAAACCGGGGCCCAAGGCAACCGATG 111
R2HP3'_19   CACCTGATTCTTGAGTAGGCCCCGGGAAACCGGGGCCCAAGGCAACCGATG 111
R2HP3'_20   CACCTGATTCTTGAGTAGGCCCCGGGAAACCGGGGCCCAAGGCAACCGATG 111
            **** *************       ****       ***************
                                 Hairpin
```
