# Supplementary material for: Target Site Recognition by a Diversity-Generating Retroelement
Source: PLoS Genet. 2011 Dec 15;7(12):e1002414. doi: 10.1371/journal.pgen.1002414 (PMC3240598; doi:10.1371/journal.pgen.1002414)
Supplement: Figure S4 — Sequence analysis of tropism switching products of phage BPP-1ΔATR with WT hairpin/cruciform structures. Sequences from the beginning of VR to the start codon of avd of five progeny phages with switched tropisms were aligned with the corresponding region of the predicted WT homing product lacking adenine mutagenesis (TR99VR). The hairpin region is underlined in red and adenine mutagenesis is observed in all five progeny phages with switched tropisms. (PDF) [file pgen.1002414.s004.pdf]

```
TR99VR   CGCTGCTGCGCTATTCGGCGGCAACTGGAACAACACGTCGAACTCGGGTTCTCGCGCTGC 60
wtTSP1   CGCTGCTGCGCTATTCGGCGGCGCCTGGAGCAACACGTCGAGCTCGGGTTCTCGCGCTGC 60
wtTSP2   CGCTGCTGCGCTATTCGGCGGCTCCTGGAGCAACACGTCGTACTCGGGTTCTCGCGCTGC 60
wtTSP3   CGCTGCTGCGCTATTCGGCGGCTCCTGGAGCAACACGTCGAACTCGGGTTCTCGCGCTGC 60
wtTSP4   CGCTGCTGCGCTGTTCGGCGGCTCCTGGAGCAACACGTCGAACTCGGGTTCTCGCGCTGC 60
wtTSP5   CGCTGCTGCGCTATTCGGCGGCTCCTGGAGCAACACGTCGAACTCGGGTTCTCGCGCTGC 60
         ************ *********  ***** **********  ******************
                                       VR

TR99VR   GAACTGGAACAACGGGCCGTCGAACTCGAACGCGAACATCGGGGCGCGCGGCGTCTGTGA 120
wtTSP1   GTACTGGCACAGCGGGCCGTCGTACTCGTTCGCGTTCTTCGGGGCGCGCGGCGTCTGTGA 120
wtTSP2   GTACTGGAGCTACGGGCCGTCGCTCTCGCTCGCGTTCATCGGGGCGCGCGGCGTCTGTGA 120
wtTSP3   GTACTGGTACAACGGGCCGTCGAACTCGGCCGCGTACTTCGGGGCGCGCGGCGTCTGTGA 120
wtTSP4   GTACTGGAGCGCCGGGCCGTCGTTCTCGTTCGCGTTCTTCGGGGCGCGCGGCGTCTGTGA 120
wtTSP5   GTACTGGAACTACGGGCCGTCGTACTCGAACGCGTACATCGGGGCGCGCGGCGTCTGTGA 120
         * *****  *  **********  ****  ****  * **********************
                                       VR

TR99VR   CCACCTGATTCTTGAGTAGCGGGGCCGAAAGGCCCCGCCAAGGCAACCGATG 172
wtTSP1   CCACCTGATTCTTGAGTAGCGGGGCCGAAAGGCCCCGCCAAGGCAACCGATG 172
wtTSP2   CCACCTGATTCTTGAGTAGCGGGGCCGAAAGGCCCCGCCAAGGCAACCGATG 172
wtTSP3   CCACCTGATTCTTGAGTAGCGGGGCCGAAAGGCCCCGCCAAGGCAACCGATG 172
wtTSP4   CCACCTGATTCTTGAGTAGCGGGGCCGAAAGGCCCCGCCAAGGCAACCGATG 172
wtTSP5   CCACCTGATTCTTGAGTAGCGGGGCCGAAAGGCCCCGCCAAGGCAACCGATG 172
         ****************************************************
              VR               WT Hairpin
```
