# Supplementary material for: Target Site Recognition by a Diversity-Generating Retroelement
Source: PLoS Genet. 2011 Dec 15;7(12):e1002414. doi: 10.1371/journal.pgen.1002414 (PMC3240598; doi:10.1371/journal.pgen.1002414)
Supplement: Figure S5 — Sequence analysis of tropism switching products of phage BPP-1ΔATR StMut. Sequences from the beginning of VR to the start codon of avd of five tropism-switched progeny phages of recipient StMut were aligned with the corresponding region of the predicted WT homing product lacking adenine mutagenesis (TR99VR). The hairpin region is underlined in red to show disruption of the structure. Adenine mutagenesis is observed in all five phage tropism switching products. (PDF) [file pgen.1002414.s005.pdf]

```
TR99VR      CGCTGCTGCGCTATTCGGCGGCAACTGGAACAACACGTCGAACTCGGGTTCTCGCGCTGC 60
MutTSP6     CGCTGCTGCGCTATTCGGCGGCAGCTGGAGCAACACGTCGAACTCGGGTTCTCGCGCTGC 60
MutTSP7     CGCTGCTGCGCTATTCGGCGGCTCCTGGAGCAACTCGTCGGCCTCGGGTTCTCGCGCTGC 60
MutTSP8     CGCTGCTGCGCTATTCGGCGGCGCCTGGAGCAACACGTCGAACTCGGGTTCTCGCGCTGC 60
MutTSP9     CGCTGCTGCGCTATTCGGCGGCAGCTGGAGCAACACGTCGAACTCGGGTTCTCGCGCTGC 60
MutTSP10    CGCTGCTGCGCTATTCGGCGGCGCCTGGAGCAACACGTCGTCCTCGGGTTCTCGCGCTGC 60
            **********************  ***** **** *****  ******************
                                       VR

TR99VR      GAACTGGAACAACGGGCCGTCGAACTCGAACGCGAACATCGGGGCGCGCGGCGTCTGTGA 120
MutTSP6     GTACTGGTACTACGGGCCGTCGTACTCGAGCGCGTACCTCGGGGCGCGCGGCGTCTGTGA 120
MutTSP7     GTACTGGGACTACGGGCCGTCGACCTCGTGCGCGTACATCGGGGCGCGCGGCGTCTGTGA 120
MutTSP8     GTACTGGAGCTACGGGCCGTCGAGCTCGGGCGCGTACTTCGGGGCGCGCGGCGTCTGTGA 120
MutTSP9     GTACTGGAGCAGCGGGCCGTCGAACTCGTTCGCGTTCTTCGGGGCGCGCGGCGTCTGTGA 120
MutTSP10    GGCCTGGAGCTACGGGCCGTCGGGCTCGGGCGCGTTCATCGGGGCGCGCGGCGTCTGTGA 120
            *  ****  *  **********  ****  ****  * **********************
                                       VR

TR99VR      CCACCTGATTCTTGAGTAGCGGGGCCGAAAGGCCCCGCCAAGGCAACCGATG 172
MutTSP6     CCACCTGATTCTTGAGTAGCGGGGCCGAAACCGGGGCCCAAGGCAACCGATG 172
MutTSP7     CCACCTGATTCTTGAGTAGCGGGGCCGAAACCGGGGCCCAAGGCAACCGATG 172
MutTSP8     CCACCTGATTCTTGAGTAGCGGGGCCGAAACCGGGGCCCAAGGCAACCGATG 172
MutTSP9     CCACCTGATTCTTGAGTAGCGGGGCCGAAACCGGGGCCCAAGGCAACCGATG 172
MutTSP10    CCACCTGATTCTTGAGTAGCGGGGCCGAAACCGGGGCCCAAGGCAACCGATG 172
            ******************************       ***************
                  VR               Hairpin Region
```
