# Supplementary material for: Target Site Recognition by a Diversity-Generating Retroelement
Source: PLoS Genet. 2011 Dec 15;7(12):e1002414. doi: 10.1371/journal.pgen.1002414 (PMC3240598; doi:10.1371/journal.pgen.1002414)
Supplement: Figure S6 — Sequence analysis of tropism switching products of phage BPP-1ΔATR StRev. Sequences from the beginning of VR to the start codon of avd of five tropism-switched progeny phages of recipient StRev were aligned with the corresponding region of the predicted WT homing product lacking adenine mutagenesis (TR99VR). The hairpin region is underlined in red to show complementary changes. Adenine mutagenesis is observed in all five phage tropism switching products. (PDF) [file pgen.1002414.s006.pdf]

```
TR99VR     CGCTGCTGCGCTATTCGGCGGCAACTGGAACAACACGTCGAACTCGGGTTCTCGCGCTGC 60
RevTSP11   CGCTGCTGCGCTATTCGGCGGCGCCTGGGCCAACACGTCGAACTCGGGTTCTCGCGCTGC 60
RevTSP12   CGCTGCTGCGCTATTCGGCGGCGCCTGGAGCAACACGTCGTACTCGGGTTCTCGCGCTGC 60
RevTSP13   CGCTGCTGCGCTATTCGGCGGCTCCTGGAACAACACGTCGAACTCGGGTTCTCGCGCTGC 60
RevTSP14   CGCTGCTGCGCTATTCGGCGGCTCCTGGAGCAACACGTCGAGCTCGGGTTCTCGCGCTGC 60
RevTSP15   CGCTGCTGCGCTATTCGGCGGCGCCTGGCTCCTCACGTCGTTCTCGGGTTCTCGCGCTGC 60
           **********************  ****  *  *******  ******************
                                       VR

TR99VR     GAACTGGAACAACGGGCCGTCGAACTCGAACGCGAACATCGGGGCGCGCGGCGTCTGTGA 120
RevTSP11   GTACTGGAGCTACGGGCCGTCGTCCTCGTACGCGTACATCGGGGCGCGCGGCGTCTGTGA 120
RevTSP12   GTACTGGAACAACGGGCCGTCGTTCTCGTTCGCGTTCTTCGGGGCGCGCGGCGTCTGTGA 120
RevTSP13   GTACTGGGGCCTCGGGCCGTCGAACTCGTACGCGTACATCGGGGCGCGCGGCGTCTGTGA 120
RevTSP14   GTACTGGAGCTACGGGCCGTCGGCCTCGAACGCGTACCTCGGGGCGCGCGGCGTCTGTGA 120
RevTSP15   GTACTGGAGCTACGGGCCGTCGAACTCGCACGCGTACATCGGGGCGCGCGGCGTCTGTGA 120
           * *****  *  **********  ****  ****  * **********************
                                       VR

TR99VR     CCACCTGATTCTTGAGTAGCGGGGCCGAAAGGCCCCGCCAAGGCAACCGATG 172
RevTSP11   CCACCTGATTCTTGAGTAGGCCCCGGGAAACCGGGGCCCAAGGCAACCGATG 172
RevTSP12   CCACCTGATTCTTGAGTAGGCCCCGGGAAACCGGGGCCCAAGGCAACCGATG 172
RevTSP13   CCACCTGATTCTTGAGTAGGCCCCGGGAAACCGGGGCCCAAGGCAACCGATG 172
RevTSP14   CCACCTGATTCTTGAGTAGGCCCCGGGAAACCGGGGCCCAAGGCAACCGATG 172
RevTSP15   CCACCTGATTCTTGAGTAGGCCCCGGGAAACCGGGGCCCAAGGCAACCGATG 172
           *******************       ****      ****************
                 VR                Hairpin StemRev
```
