# Supplementary material for: Target Site Recognition by a Diversity-Generating Retroelement
Source: PLoS Genet. 2011 Dec 15;7(12):e1002414. doi: 10.1371/journal.pgen.1002414 (PMC3240598; doi:10.1371/journal.pgen.1002414)
Supplement: Figure S7 — Analysis of homing products of recipient VRInv. (A) PCR detection strategy for homing products of recipient VRInv and regions of the products aligned in (B) and (C). Primer annealing sites are indicated as small horizontal arrows. (B) Alignment of homing products of recipient VRInv from the 5′ end of VR to the end of the TG2 tag with the corresponding region of the predicted homing product lacking adenine mutagenesis (VR5′end). Adenine mutagenesis is observed in 5/10 cloned homing products. (C) Alignment of homing products of recipient VRInv from the beginning of TG2 to the end of VR with the corresponding region of the predicted WT homing product lacking adenine mutagenesis (VR3′end). Adenine mutagenesis is observed in 2/9 cloned homing products. (PDF) [file pgen.1002414.s007.pdf]

A

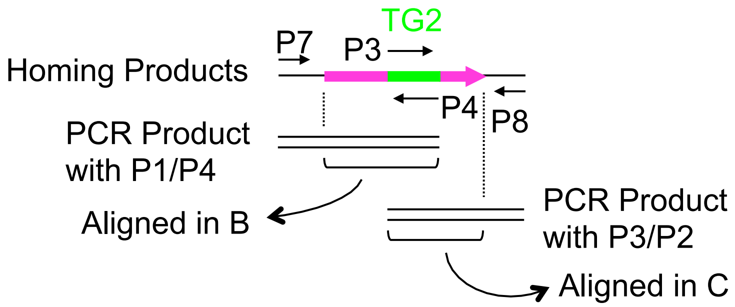

B

```
VR5'end      CGCTGCTGCGCTATTCGGCGGCAGATCTGTCTGCGTTTGTGTTCCTGTGCTAAGCTT 57
Inv5'HP01    CGCTGCTGCGCGTTTCGGCGGCAGATCTGTCTGCGTTTGTGTTCCTGTGCTAAGCTT 57
Inv5'HP02    CGCTGCTGCGCTATTCGGCGGCGGCTCTGTCTGCGTTTGTGTTCCTGTGCTAAGCTT 57
Inv5'HP03    CGCTGCTGCGCTATTCGGCGGCTGCTCTGTCTGCGTTTGTGTTCCTGTGCTAAGCTT 57
Inv5'HP04    CGCTGCTGCGCTATTCGGCGGCAGATCTGTCTGCGTTTGTGTTCCTGTGCTAAGCTT 57
Inv5'HP05    CGCTGCTGCGCTATTCGGCGGCAGATCTGTCTGCGTTTGTGTTCCTGTGCTAAGCTT 57
Inv5'HP06    CGCTGCTGCGCGATTCGGCGGCAGATCCGTCTGCGTTTGTGTTCCTGTGCTAAGCTT 57
Inv5'HP07    CGCTGCTGCGCTATTCGGCGGCGGTCTGTCTGCGTTTGTGTTCCTGTGCTAAGCTT 57
Inv5'HP08    CGCTGCTGCGCGATTCGGCGGCAGTTCTGTCTGCGTTTGT-TTCCTGTGCTAAGCTT 56
Inv5'HP09    CGCTGCTGCGCTATTCGGCGGCAGATCTGTCTGCGTTTGTGTTCCTGTGCTAAGCTT 57
Inv5'HP10    CGCTGCTGCGCTATTCGGCGGCAGATCTGTCTGCGTTTGTGTTCCTGTGCTAAGCTT 57
             ***********  ********* *  **  ************ ****************
                                                  P4
```

C

```
VR3'end      TCTAGATCTGTCTGCGTTTGTGTTCCTGTGCTAGCCATCGGGGCGCGCGGCGTCTGTGAC 60
Inv3'HP01    TCTAGATCTGTCTGCGTTTGTGTTCCTGTGCTAGCCATCGGGGCGCGCGGCGTCTGTGAC 60
Inv3'HP02    TCTAGATCTGTCTGCGTTTGTGTTCCTGTGCTAGCCATCGGGGCGCGCGGCGTCTGTGAC 60
Inv3'HP03    TCTAGATCTGTCTGCGTTTGTGTTCCTGTGCTAGCCCTCGGGGCGCGCGGCGTCTGTGAC 60
Inv3'HP04    TCTAGATCTGTCTGCGTTTGTGTTCCTGTGCTAGCCATCGGGGCGCGCGGCGTCTGTGAC 60
Inv3'HP05    TCTAGATCTGTCTGCGTTTGTGTTCCTGTGCTAGCCATCGGGGCGCGCGGCGTCTGTGAC 60
Inv3'HP06    TCTAGATCTGTCTGCGTTTGTGTTCCTGTGCTAGCCATCGGGGCGCGCGGCGTCTGTGAC 60
Inv3'HP07    TCTAGATCTGTCTGCGTTTGTGTTCCTGTGCTAGCCTTCGGGGCGCGCGGCGTCTGTGAC 60
Inv3'HP08    TCTAGATCTGTCTGCGTTTGTGTTCCTGTGCTAGCCATCGGGGCGCGCGGCGTCTGTGAC 60
Inv3'HP09    TCTAGATCTGTCTGCGTTTGTGTTCCTGTGCTAGCCAGCGGGGCGCGCGGCGTCTGTGAC 60
             ************************************  **********************
                          P3                                G/C

VR3'end      CACCTGATTCTTG 73
Inv3'HP01    CACCTGATTCTTG 73
Inv3'HP02    CACCTGATTCTTG 73
Inv3'HP03    CACCTGATTCTTG 73
Inv3'HP04    CACCTGATTCTTG 73
Inv3'HP05    CACCTGATTCTTG 73
Inv3'HP06    CACCTGATTCTTG 73
Inv3'HP07    CACCTGATTCTTG 73
Inv3'HP08    CACCTGATTCTTG 73
Inv3'HP09    CACCTGATTCTTG 73
             *************
```
