# Supplementary figures and images for: Target Site Recognition by a Diversity-Generating Retroelement
Source: PLoS Genet. 2011 Dec 15;7(12):e1002414. doi: 10.1371/journal.pgen.1002414 (PMC3240598; doi:10.1371/journal.pgen.1002414)

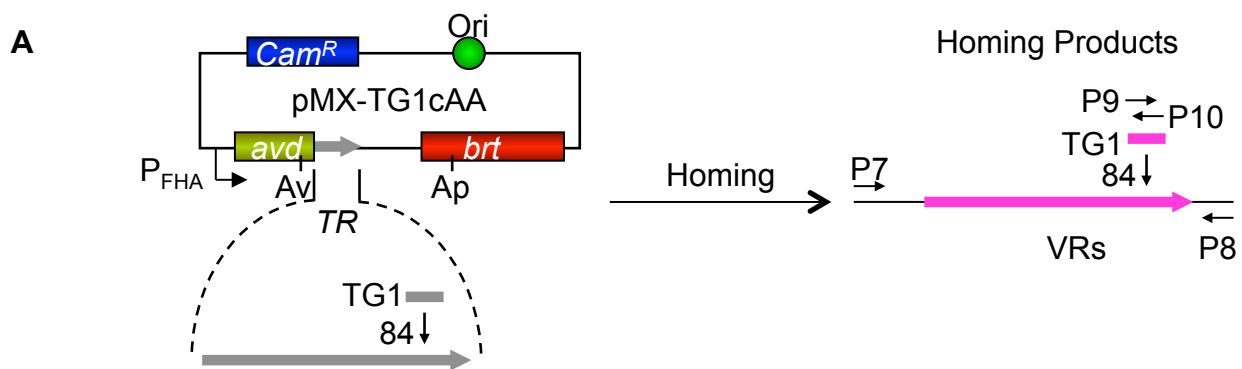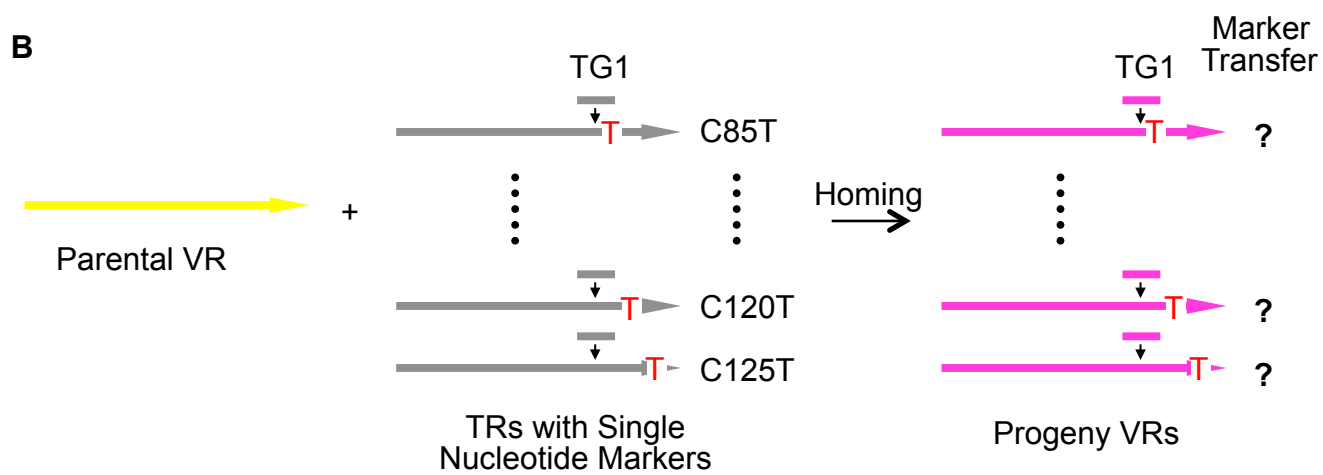

Supplement: Figure S8 — Outline of marker coconversion assay with recipient phage BPP-1ΔATRSpI6. (A) PCR-based DGR homing assays with marked donor plasmids. Markers were introduced into plasmid pMX-TG1cAA [4]. The TR contains a 36 bp insert (TG1) at position 84. Grey and pink arrows represent TR and progeny VRs, respectively. Small horizontal arrows indicate primers used for homing assays: P7 and P8 are sense- and antisense-strand primers annealing upstream and downstream of VR, respectively; P9 and P10 (Table 1) are sense- and antisense-strand primers, respectively, that anneal to TG1. CamR, chloramphenicol resistance gene. (B) Schematic of coconversion experiments to determine 3′ marker transfer boundaries. Single C to T markers downstream of the TG1 tag in donor TRs are indicated and the constructs have been previously described [4]. Markers (red T residues) are transferred to VR only if they are located between the TR positions corresponding to 3′ and 5′ cDNA integration sites in VR. (PDF) [file pgen.1002414.s008.pdf]
