# Supplementary material for: Target Site Recognition by a Diversity-Generating Retroelement
Source: PLoS Genet. 2011 Dec 15;7(12):e1002414. doi: 10.1371/journal.pgen.1002414 (PMC3240598; doi:10.1371/journal.pgen.1002414)
Supplement: Figure S9 — Sequence analysis of replicating phage KanR targeting products with the pMX-Km1 donor. Sequences from the beginning of VR-KanS to the end of the hairpin structure were aligned with the corresponding region of the predicted KanR targeting product lacking adenine mutagenesis (KmHP). The targeting assay was carried out with BPP-1ΔATR*KanS single-cycle lytic infection of RB50 cells transformed with donor plasmid pMX-Km1. Progeny phages were used to generate lysogens in RB50 cells, which were analyzed on plates with and without kanamycin to determine the efficiency of KanR targeting. KanR clones were sequenced to verify regeneration of full-length KanR genes. Adenine mutagenesis is observed in 7/10 clones. (PDF) [file pgen.1002414.s009.pdf]

```
KmHP     CGCTTGCAGTTTCATTTGATGCTCGATGAGTTTTTCTAATAAGCTAGCCATCGGGGCGCG 60
RP1-01   CGCTTGCAGTTCCATTTGATGCTCGATGAGTTTTTCTAATAAGCTGGCCGTCGGGGCGCG 60
RP1-02   CGCTTGCAGTTTCATTTGATGCTCGATGAGTTTTTCTAATAAGCTAGCCATCGGGGCGCG 60
RP1-03   CGCTTGCAGTTTCATTTGATGCTCGATGAGTTTTTCTAGTAAGCTAGCCATCGGGGCGCG 60
RP1-04   CGCTTGCAGTTTCATTTGATGCTCGATGAGTTTTTCTAATAAGCTGGCCATCGGGGCGCG 60
RP1-05   CGCTTGCAGTTTCATTTGATGCTCGATGAGTTTTTCTAATAAGCTAGCCATCGGGGCGCG 60
RP1-06   CGCTTGCAGTTTCATTTGATGCTCGATGAGTTTTTCTAATAAGCTAGCCATCGGGGCGCG 60
RP1-07   CGCTTGCAGTTTCATTTGGTGCTCGATGAGTTTTTCTAGTAAGCTCGCCATCGGGGCGCG 60
RP1-08   CGCTTGCAGTTTCATTTGATGCTCGATGAGTTTTTCTAGTAAGCTGGCCATCGGGGCGCG 60
RP1-09   CGCTTGCAGTTTCATTTGATGCTCGATGAGTTTTTCTAATAGGCTGGCCATCGGGGCGCG 60
RP1-10   CGCTTGCAGTTTCATTTGTTGCTCGATGAGTTTTTCTAATAAGCTAGCCGTCGGGGCGCG 60
         *********** ****** ******************* ** *** *** **********
               Regenerated KanR 3' end                        GC

KmHP     CGGCGTCTGTGACCACCTGATTCTTGAGTAGCGGGGCCGAAAGGCCCCGC 110
RP1-01   CGGCGTCTGTGACCACCTGATTCTTGAGTAGCGGGGCCGAAAGGCCCCGC 110
RP1-02   CGGCGTCTGTGACCACCTGATTCTTGAGTAGCGGGGCCGAAAGGCCCCGC 110
RP1-03   CGGCGTCTGTGACCACCTGATTCTTGAGTAGCGGGGCCGAAAGGCCCCGC 110
RP1-04   CGGCGTCTGTGACCACCTGATTCTTGAGTAGCGGGGCCGAAAGGCCCCGC 110
RP1-05   CGGCGTCTGTGACCACCTGATTCTTGAGTAGCGGGGCCGAAAGGCCCCGC 110
RP1-06   CGGCGTCTGTGACCACCTGATTCTTGAGTAGCGGGGCCGAAAGGCCCCGC 110
RP1-07   CGGCGTCTGTGACCACCTGATTCTTGAGTAGCGGGGCCGAAAGGCCCCGC 110
RP1-08   CGGCGTCTGTGACCACCTGATTCTTGAGTAGCGGGGCCGAAAGGCCCCGC 110
RP1-09   CGGCGTCTGTGACCACCTGATTCTTGAGTAGCGGGGCCGAAAGGCCCCGC 110
RP1-10   CGGCGTCTGTGACCACCTGATTCTTGAGTAGCGGGGCCGAAAGGCCCCGC 110
         **************************************************
                                               WT Hairpin
```
