# Supplementary material for: Target Site Recognition by a Diversity-Generating Retroelement
Source: PLoS Genet. 2011 Dec 15;7(12):e1002414. doi: 10.1371/journal.pgen.1002414 (PMC3240598; doi:10.1371/journal.pgen.1002414)
Supplement: Figure S10 — Sequence analysis of replicating phage KanR targeting products with the pMX-Km2 donor. Sequences from the beginning of VR-KanS to the end of the hairpin structure were aligned with the corresponding region of the predicted KanR targeting product lacking adenine mutagenesis (KmHP). The targeting assay was carried out with BPP-1ΔATR*KanS single-cycle lytic infection of RB50 cells transformed with donor plasmid pMX-Km2. RB50 cells were lysogenized with progeny phages and subsequently analyzed on plates with and without kanamycin to determine the efficiency of KanR targeting. KanR clones were sequenced to verify regeneration of full-length KanR genes. Adenine mutagenesis is observed in 7/11 clones. (PDF) [file pgen.1002414.s010.pdf]

```
KmHP     CGCTTGCAGTTTCATTTGATGCTCGATGAGTTTTTCTAATAAGCTAGCCATCGGGGCGCG 60
RP2-01   CGCTTGCAGTTTCATTTGATGCTCGATGAGTTTTTCTAATAAGCTAGCCTTCGGGGCGCG 60
RP2-02   CGCTTGCAGTTTCATTTGATGCTCGATGAGTTTTTCTAATGGGCTAGCCATCGGGGCGCG 60
RP2-03   CGCTTGCAGTTTCATTTGATGCTCGATGAGTTTTTCTAATAAGCTAGCCATCGGGGCGCG 60
RP2-04   CGCTTGCAGTTTCATTTGATGCTCGATGAGTTTTTCTAATAAGCTAGCCATCGGGGCGCG 60
RP2-05   CGCTTGCAGTTTCATTTGATGCTCGATGAGTTTTTCTAATAGGCTAGCCATCGGGGCGCG 60
RP2-06   CGCTTGCAGTTTCATTTGGTGCTCGATGAGTTTTTCTAATAAGCTAGGCCTCGGGGCGCG 60
RP2-07   CGCTTGCAGTTTCATTTGCTGCTCGATGAGTTTTTCTAATGGGCTGGCCGTCGGGGCGCG 60
RP2-08   CGCTTGCAGTTTCATTTGATGCTCGATGAGTTTTTCTAATAAGCTAGCCATCGGGGCGCG 60
RP2-09   CGCTTGCAGTTTCATTTGATGCTCGATGAGTTTTTCTAATAAGCTAGCCATCGGGGCGCG 60
RP2-10   CGCTTGCAGTTTCATTTGATGCTCGATGAGCTTTTCTAGTAAGCTGGCCATCGGGGCGCG 60
RP2-11   CGCTTGCAGTTTCATTTGATGCTCGATGAGTTTTTCTAGTAAGCTAGCCATCGGGGCGCG 60
         ****************** *********** ******* *   *** * * **********
               Regenerated KanR 3' end                          GC

KmHP     CGGCGTCTGTGACCACCTGATTCTTGAGTAGCGGGGCCGAAAGGCCCCGC 110
RP2-01   CGGCGTCTGTGACCACCTGATTCTTGAGTAGCGGGGCCGAAAGGCCCCGC 110
RP2-02   CGGCGTCTGTGACCACCTGATTCTTGAGTAGCGGGGCCGAAAGGCCCCGC 110
RP2-03   CGGCGTCTGTGACCACCTGATTCTTGAGTAGCGGGGCCGAAAGGCCCCGC 110
RP2-04   CGGCGTCTGTGACCACCTGATTCTTGAGTAGCGGGGCCGAAAGGCCCCGC 110
RP2-05   CGGCGTCTGTGACCACCTGATTCTTGAGTAGCGGGGCCGAAAGGCCCCGC 110
RP2-06   CGGCGTCTGTGACCACCTGATTCTTGAGTAGCGGGGCCGAAAGGCCCCGC 110
RP2-07   CGGCGTCTGTGACCACCTGATTCTTGAGTAGCGGGGCCGAAAGGCCCCGC 110
RP2-08   CGGCGTCTGTGACCACCTGATTCTTGAGTAGCGGGGCCGAAAGGCCCCGC 110
RP2-09   CGGCGTCTGTGACCACCTGATTCTTGAGTAGCGGGGCCGAAAGGCCCCGC 110
RP2-10   CGGCGTCTGTGACCACCTGATTCTTGAGTAGCGGGGCCGAAAGGCCCCGC 110
RP2-11   CGGCGTCTGTGACCACCTGATTCTTGAGTAGCGGGGCCGAAAGGCCCCGC 110
         **************************************************
                                              WT Hairpin
```
