# Supplementary material for: Target Site Recognition by a Diversity-Generating Retroelement
Source: PLoS Genet. 2011 Dec 15;7(12):e1002414. doi: 10.1371/journal.pgen.1002414 (PMC3240598; doi:10.1371/journal.pgen.1002414)
Supplement: Figure S11 — Sequence analysis of prophage KanR targeting products with the pMX-Km1 donor. Sequences from the beginning of VR-KanS to the end of the hairpin structure were aligned with the corresponding region of the predicted KanR retargeting product lacking adenine mutagenesis (KmHP). The targeting assay was carried out in BPP-1ΔATR*KanS lysogen cells transformed with donor plasmid pMX-Km1. Resulting cells were analyzed on plates with and without kanamycin to determine the efficiency of KanR targeting. KanR clones were then sequenced to verify regeneration of full-length KanR genes. Adenine mutagenesis is observed in 13/16 clones. (PDF) [file pgen.1002414.s011.pdf]

```
KmHP     CGCTTGCAGTTTCATTTGATGCTCGATGAGTTTTTCTAATAAGCTAGCCATCGGGGCGCG 60
LY1-01   CGCTTGCAGTTTCATTTGATGCTCGATGAGTTTTTCTAGTGAGCTAGCCATCGGGGCGCG 60
LY1-02   CGCTTGCAGTTTCATTTGATGCTCGGTGAGTTTTTCTAATAAGCTAGCCATCGGGGCGCG 60
LY1-03   CGCTTGCAGTTTCATTTGATGCTCGGTGAGTTTTTCTAATGGGCTAGCCATCGGGGCGCG 60
LY1-04   CGCTTGCAGTTTCATTTGTTGCTCGATGAGTTTTTCTAATGAGCTAGCCATCGGGGCGCG 60
LY1-05   CGCTTGCAGTTTCATTTGATGCTCGGTGAGTTTTTCTAATAAGCTAGCCTTCGGGGCGCG 60
LY1-06   CGCTTGCAGTTTCATTTGATGCTCGATGAGTTTTTCTAATGAGCTAGCCATCGGGGCGCG 60
LY1-07   CGCTTGCAGTTTCATTTGATGCTCGATGAGTTTTTCTAATGAGCTAGCCATCGGGGCGCG 60
LY1-08   CGCTTGCAGTTTCATTTGTTGCTCGATGAGTTTTTCTAGTAAGCTAGCCCTCGGGGCGCG 60
LY1-09   CGCTTGCAGTTTCATTTGATGCTCGATGAGTTTTTCTAGTAAGCTAGCCATCGGGGCGCG 60
LY1-10   CGCTTGCAGTTTCATTTGATGCTCGATGAGTTTTTCTAATAAGCTAGCCATCGGGGCGCG 60
LY1-11   CGCTTGCAGTTTCATTTGTTGCTCGATGAGTTTTTCTAATAAGCTAGCCATCGGGGCGCG 60
LY1-12   CGCTTGCAGTTTCATTTGATGCTCGGTGAGTTTTTCTAATAAGCTAGCCATCGGGGCGCG 60
LY1-13   CGCTTGCAGTTTCATTTGATGCTCGATGAGTTTTTCTAGTAAGCTAGCCATCGGGGCGCG 60
LY1-14   CGCTTGCAGTTTCATTTGATGCTCGATGAGTTTTTCTAATAAGCTAGCCATCGGGGCGCG 60
LY1-15   CGCTTGCAGTTTCATTTGATGCTCGATGAGTTTTTCTAATAAGCTAGCCATCGGGGCGCG 60
LY1-16   CGCTTGCAGTTTCATTTGATGCTCGATGAGTTTTTCTAATAAGCTAGCCGTCGGGGCGCG 60
         ****************** ****** ************ *  ******* **********
               Regenerated KanR 3' end                         GC

KmHP     CGGCGTCTGTGACCACCTGATTCTTGAGTAGCGGGGCCGAAAGGCCCCGC 110
LY1-01   CGGCGTCTGTGACCACCTGATTCTTGAGTAGCGGGGCCGAAAGGCCCCGC 110
LY1-02   CGGCGTCTGTGACCACCTGATTCTTGAGTAGCGGGGCCGAAAGGCCCCGC 110
LY1-03   CGGCGTCTGTGACCACCTGATTCTTGAGTAGCGGGGCCGAAAGGCCCCGC 110
LY1-04   CGGCGTCTGTGACCACCTGATTCTTGAGTAGCGGGGCCGAAAGGCCCCGC 110
LY1-05   CGGCGTCTGTGACCACCTGATTCTTGAGTAGCGGGGCCGAAAGGCCCCGC 110
LY1-06   CGGCGTCTGTGACCACCTGATTCTTGAGTAGCGGGGCCGAAAGGCCCCGC 110
LY1-07   CGGCGTCTGTGACCACCTGATTCTTGAGTAGCGGGGCCGAAAGGCCCCGC 110
LY1-08   CGGCGTCTGTGACCACCTGATTCTTGAGTAGCGGGGCCGAAAGGCCCCGC 110
LY1-09   CGGCGTCTGTGACCACCTGATTCTTGAGTAGCGGGGCCGAAAGGCCCCGC 110
LY1-10   CGGCGTCTGTGACCACCTGATTCTTGAGTAGCGGGGCCGAAAGGCCCCGC 110
LY1-11   CGGCGTCTGTGACCACCTGATTCTTGAGTAGCGGGGCCGAAAGGCCCCGC 110
LY1-12   CGGCGTCTGTGACCACCTGATTCTTGAGTAGCGGGGCCGAAAGGCCCCGC 110
LY1-13   CGGCGTCTGTGACCACCTGATTCTTGAGTAGCGGGGCCGAAAGGCCCCGC 110
LY1-14   CGGCGTCTGTGACCACCTGATTCTTGAGTAGCGGGGCCGAAAGGCCCCGC 110
LY1-15   CGGCGTCTGTGACCACCTGATTCTTGAGTAGCGGGGCCGAAAGGCCCCGC 110
LY1-16   CGGCGTCTGTGACCACCTGATTCTTGAGTAGCGGGGCCGAAAGGCCCCGC 110
         **************************************************
                                                WT Hairpin
```
