# Supplementary material for: Target Site Recognition by a Diversity-Generating Retroelement
Source: PLoS Genet. 2011 Dec 15;7(12):e1002414. doi: 10.1371/journal.pgen.1002414 (PMC3240598; doi:10.1371/journal.pgen.1002414)
Supplement: Figure S12 — Sequence analysis of prophage KanR targeting products with the pMX-Km2 donor. Sequences from the beginning of VR-KanS to the end of the hairpin structure were aligned with the corresponding region of the predicted KanR retargeting product lacking adenine mutagenesis (KmHP). The targeting assay was carried out in BPP-1ΔATR*KanS lysogen cells transformed with donor plasmid pMX-Km2. Resulting cells were plated on plates with and without kanamycin to determine the efficiency of KanR targeting. KanR clones were sequenced to confirm regeneration of full-length KanR genes. Adenine mutagenesis is observed in 13/16 clones. (PDF) [file pgen.1002414.s012.pdf]

```
KmHP    CGCTTGCAGTTTCATTTGATGCTCGATGAGTTTTTCTAATAAGCTAGCCATCGGGGCGCG 60
LY2-01  CGCTTGCAGTTTCATTTGATGCTCGATGAGTTTGTCTAATAGGCTAGCCATCGGGGCGCG 60
LY2-02  CGCTTGCAGTTTCATTTGATGCTCGATGAGTTTTTCTAGTAAGCTAGCCATCGGGGCGCG 60
LY2-03  CGCTTGCAGTTTCATTTGATGCTCGATGAGTTTTTCTAATAAGCTAGCCATCGGGGCGCG 60
LY2-04  CGCTTGCAGTTTCATTTGATGCTCGATGAGTTTTTCTAATGAGCTAGCCATCGGGGCGCG 60
LY2-05  CGCTTGCAGTTTCATTTGATGCTCGATGAGTTTTTCTAGTAAGCTAGCCATCGGGGCGCG 60
LY2-06  CGCTTGCAGTTTCATTTGATGCTCGCTGAGTTTTTCTAGTAAGCTAGCCATCGGGGCGCG 60
LY2-07  CGCTTGCAGTTTCATTTGATGCTCGATGAGTTTTTCTAATAAGCTAGCCATCGGGGCGCG 60
LY2-08  CGCTTGCAGTTTCATTTGATGCTCGATGAGTTTTTCTAATGGGCTAGCCATCGGGGCGCG 60
LY2-09  CGCTTGCAGTTTCATTTGATGCTCGATGAGTTTTTCTAGTGGGCTGGCCATCGGGGCGCG 60
LY2-10  CGCTTGCAGTTTCATTTGTTGCTCGATGAGTTTTTCTAATAAGCTAGCCATCGGGGCGCG 60
LY2-11  CGCTTGCAGTTTCATTTGATGCTCGATGAGTTTTTCTGATAAGCTAGCCATCGGGGCGCG 60
LY2-12  CGCTTGCAGTTTCATTTGATGCTCGATGAGTTTTTCTAGTAAGCTAGCCATCGGGGCGCG 60
LY2-13  CGCTTGCAGTTTCATTTGATGCTCGGTGAGTTTTTCTAATGAGCTAGCCATCGGGGCGCG 60
LY2-14  CGCTTGCAGTTTCATTTGATGCTCGATGAGTTTTTCTAGTAAGCTAGCCCTCGGGGCGCG 60
LY2-15  CGCTTGCAGTTTCATTTGTTGCTCGATGAGTTTTTCTAATAAGCTAGCCATCGGGGCGCG 60
LY2-16  CGCTTGCAGTTTCATTTGATGCTCGATGAGTTTTTCTAATAAGCTAGCCATCGGGGCGCG 60
        ****************** ****** ******* ***   *   *** *** **********
```

Regenerated *Kan*^{R} 3′ end — G/C

```
KmHP    CGGCGTCTGTGACCACCTGATTCTTGAGTAGCGGGGCCGAAAGGCCCCGC 110
LY2-01  CGGCGTCTGTGACCACCTGATTCTTGAGTAGCGGGGCCGAAAGGCCCCGC 110
LY2-02  CGGCGTCTGTGACCACCTGATTCTTGAGTAGCGGGGCCGAAAGGCCCCGC 110
LY2-03  CGGCGTCTGTGACCACCTGATTCTTGAGTAGCGGGGCCGAAAGGCCCCGC 110
LY2-04  CGGCGTCTGTGACCACCTGATTCTTGAGTAGCGGGGCCGAAAGGCCCCGC 110
LY2-05  CGGCGTCTGTGACCACCTGATTCTTGAGTAGCGGGGCCGAAAGGCCCCGC 110
LY2-06  CGGCGTCTGTGACCACCTGATTCTTGAGTAGCGGGGCCGAAAGGCCCCGC 110
LY2-07  CGGCGTCTGTGACCACCTGATTCTTGAGTAGCGGGGCCGAAAGGCCCCGC 110
LY2-08  CGGCGTCTGTGACCACCTGATTCTTGAGTAGCGGGGCCGAAAGGCCCCGC 110
LY2-09  CGGCGTCTGTGACCACCTGATTCTTGAGTAGCGGGGCCGAAAGGCCCCGC 110
LY2-10  CGGCGTCTGTGACCACCTGATTCTTGAGTAGCGGGGCCGAAAGGCCCCGC 110
LY2-11  CGGCGTCTGTGACCACCTGATTCTTGAGTAGCGGGGCCGAAAGGCCCCGC 110
LY2-12  CGGCGTCTGTGACCACCTGATTCTTGAGTAGCGGGGCCGAAAGGCCCCGC 110
LY2-13  CGGCGTCTGTGACCACCTGATTCTTGAGTAGCGGGGCCGAAAGGCCCCGC 110
LY2-14  CGGCGTCTGTGACCACCTGATTCTTGAGTAGCGGGGCCGAAAGGCCCCGC 110
LY2-15  CGGCGTCTGTGACCACCTGATTCTTGAGTAGCGGGGCCGAAAGGCCCCGC 110
LY2-16  CGGCGTCTGTGACCACCTGATTCTTGAGTAGCGGGGCCGAAAGGCCCCGC 110
        **************************************************
```

WT Hairpin
