# Supplementary material for: Target Site Recognition by a Diversity-Generating Retroelement
Source: PLoS Genet. 2011 Dec 15;7(12):e1002414. doi: 10.1371/journal.pgen.1002414 (PMC3240598; doi:10.1371/journal.pgen.1002414)
Supplement: Figure S13 — Sequence analysis of plasmid KanR targeting products with the pMX-Km1 donor. Sequences from the beginning of VR-KanS to the end of the hairpin structure were aligned with the corresponding region of the predicted KanR targeting product lacking adenine mutagenesis (KmHP). Targeting assay was carried out in RB50 cells transformed with both recipient plasmid pHGT-KanS and donor plasmid pMX-Km1. Resulting cells were analyzed on plates with and without kanamycin to determine the efficiency of KanR targeting. KanR clones were sequenced to verify regeneration of full-length KanR genes. Adenine mutagenesis is observed in 6/7 clones. (PDF) [file pgen.1002414.s013.pdf]

```
KmHP    CGCTTGCAGTTTCATTTGATGCTCGATGAGTTTTTCTAATAAGCTAGCCATCGGGGCGCG 60
PL1-01  CGCTTGCAGTTTCATTTGATGCTCGATGAGTTTTTCTAATAAGCTAGCCATCGGGGCGCG 60
PL1-02  CGCTTGCAGTTTCATTTGATGCTCGCTGAGTTTTTCTAGTTTGCTAGCCCTCGGGGCGCG 60
PL1-03  CGCTTGCAGTTTCATTTGATGCTCGATGAGTTTTTCTAATGGGCTAGCCATCGGGGCGCG 60
PL1-04  CGCTTGCAGTTTCATTTGGTGCTCGATGAGTTTTTCTAATGAGCTAGCCATCGGGGCGCG 60
PL1-05  CGCTTGCAGTTTCATTTGATGCTCGGTGAGTTTTTCTAATCTGCTAGCCATCGGGGCGCG 60
PL1-06  CGCTTGCAGTTCCATTTGATGCTCGGTGAGTTTTTCTAATAAGCTGGCCATCGGGGCGCG 60
PL1-07  CGCTTGCAGTTTCATTTGATGCTCGTTGAGTTTTTCTAATAAGCTAGCCATCGGGGCGCG 60
        *********** ****** ****** ************ *  *** *** **********
```

Regenerated Kan^R 3' end

GC

```
KmHP    CGGCGTCTGTGACCACCTGATTCTTGAGTAGCGGGGCCGAAAGGCCCCGC 110
PL1-01  CGGCGTCTGTGACCACCTGATTCTTGAGTAGCGGGGCCGAAAGGCCCCGC 110
PL1-02  CGGCGTCTGTGACCACCTGATTCTTGAGTAGCGGGGCCGAAAGGCCCCGC 110
PL1-03  CGGCGTCTGTGACCACCTGATTCTTGAGTAGCGGGGCCGAAAGGCCCCGC 110
PL1-04  CGGCGTCTGTGACCACCTGATTCTTGAGTAGCGGGGCCGAAAGGCCCCGC 110
PL1-05  CGGCGTCTGTGACCACCTGATTCTTGAGTAGCGGGGCCGAAAGGCCCCGC 110
PL1-06  CGGCGTCTGTGACCACCTGATTCTTGAGTAGCGGGGCCGAAAGGCCCCGC 110
PL1-07  CGGCGTCTGTGACCACCTGATTCTTGAGTAGCGGGGCCGAAAGGCCCCGC 110
        **************************************************
```

WT Hairpin
