# Supplementary material for: Target Site Recognition by a Diversity-Generating Retroelement
Source: PLoS Genet. 2011 Dec 15;7(12):e1002414. doi: 10.1371/journal.pgen.1002414 (PMC3240598; doi:10.1371/journal.pgen.1002414)
Supplement: Figure S14 — Sequence analysis of plasmid KanR targeting products with the pMX-Km2 donor. Sequences from the beginning of VR-KanS to the end of the hairpin structure were aligned with the corresponding region of the predicted KanR retargeting product lacking adenine mutagenesis (KmHP). The targeting assay was carried out in RB50 cells transformed with both recipient plasmid pHGT-KanS and donor plasmid pMX-Km2. Resulting cells were plated on plates with and without kanamycin to determine the efficiency of KanR targeting. KanR clones were then sequenced to confirm regeneration of full-length KanR genes. Adenine mutagenesis is observed in 9/10 clones. (PDF) [file pgen.1002414.s014.pdf]

```
KmHP     CGCTTGCAGTTTCATTTGATGCTCGATGAGTTTTTCTAATAAGCTAGCCATCGGGGCGCG 60
PL2-01   CGCTTGCAGTTTCATTTGATGCTCGGTGAGTTTTTCTAATAAGCTAGCCATCGGGGCGCG 60
PL2-02   CGCTTGCAGTTTCATTTGATGCTCGATGAGTTTTTCTAATGGGCTAGCCATCGGGGCGCG 60
PL2-03   CGCTTGCAGTTTCATTTGATGCTCGATGAGTTTTTCTAATAAGCTAGCCATCGGGGCGCG 60
PL2-04   CGCTTGCAGTTTCATTTGATGCTCGATGAGTTTTTCTAGTAAGCTGGCCATCGGGGCGCG 60
PL2-05   CGCTTGCAGTTTCATTTGTTGCTCGATGAGTTTTTCTAATAAGCTGGCCGTCGGGGCGCG 60
PL2-06   CGCTTGCAGTTTCATTTGATGCTCGATGAGTTTTTCTGATGGGCTGGCCATCGGGGCGCG 60
PL2-07   CGCTTGCAGTTTCATTTGATGCTCGGTGAGTTTTTCTAGTAAGCTAGCCATCGGGGCGCG 60
PL2-08   CGCTTGCAGTTTCATTTGATGCTCGATGAGTTTTTCTAATGAGCTAGCCATCGGGGCGCG 60
PL2-09   CGCTTGCAGTTTCATTTGATGCTCGATGAGTTTTTCTAGTAAGGTAGCCGTCGGGGCGCG 60
PL2-10   CGCTTGCAGTTTCATTTGATGCTCGATGAGTTTTTCTAATAAGCTGGCCATCGGGGCGCG 60
         ****************** ****** ***********  *  * * *** **********
             Regenerated KanR 3' end                          GC

KmHP     CGGCGTCTGTGACCACCTGATTCTTGAGTAGCGGGGCCGAAAGGCCCCGC 110
PL2-01   CGGCGTCTGTGACCACCTGATTCTTGAGTAGCGGGGCCGAAAGGCCCCGC 110
PL2-02   CGGCGTCTGTGACCACCTGATTCTTGAGTAGCGGGGCCGAAAGGCCCCGC 110
PL2-03   CGGCGTCTGTGACCACCTGATTCTTGAGTAGCGGGGCCGAAAGGCCCCGC 110
PL2-04   CGGCGTCTGTGACCACCTGATTCTTGAGTAGCGGGGCCGAAAGGCCCCGC 110
PL2-05   CGGCGTCTGTGACCACCTGATTCTTGAGTAGCGGGGCCGAAAGGCCCCGC 110
PL2-06   CGGCGTCTGTGACCACCTGATTCTTGAGTAGCGGGGCCGAAAGGCCCCGC 110
PL2-07   CGGCGTCTGTGACCACCTGATTCTTGAGTAGCGGGGCCGAAAGGCCCCGC 110
PL2-08   CGGCGTCTGTGACCACCTGATTCTTGAGTAGCGGGGCCGAAAGGCCCCGC 110
PL2-09   CGGCGTCTGTGACCACCTGATTCTTGAGTAGCGGGGCCGAAAGGCCCCGC 110
PL2-10   CGGCGTCTGTGACCACCTGATTCTTGAGTAGCGGGGCCGAAAGGCCCCGC 110
         **************************************************
                                         WT Hairpin
```
